# Early antibiotic exposure and vaccine immune responses in preterm infants: potential sex-specific differences

**DOI:** 10.1080/19490976.2026.2694122

**Published:** 2026-06-27

**Authors:** Laura Haag, Stefanie Dietz-Ziegler, Julian Schwarz, Gabriele Kaiser, Jessica Rühle, Janine Hebel, Till Lesk, Trim Lajqi, Jennifer Müller, Ulrich Schoppmeier, Renate Fundel, Kriszta Molnar, Ingmar Fortmann, Christian F. Poets, Jana Hauke, Jürgen G. Okun, Dominik Wolff, Michael Zemlin, Christoph Härtel, Christian Gille, Natascha Köstlin-Gille

**Affiliations:** a Department of Neonatology, Tuebingen University Children's Hospital, Tuebingen, Germany; b Department of Neonatology, Heidelberg University Children's Hospital, Heidelberg, Germany; c Institute for Medical Microbiology and Hygiene, University Hospital Tuebingen, Tuebingen, Germany; d Core Facility Genomics, Medical Faculty, University Hospital Tübingen/DFG-Funded NGS Competence Center NCCT Tübingen, Tübingen, Germany; e Department of Pediatrics, University Hospital of Schleswig-Holstein, Campus Lübeck, Lübeck, Germany; f Department of Pediatrics I, Medical Faculty Heidelberg, Center for Pediatric and Adolescent Medicine, Heidelberg, Germany; g Peter L. Reichertz Institute for Medical Informatics of TU Braunschweig and Hannover Medical School, Hannover, Germany; h Department of Paediatrics, Saar University Homburg, Homburg, Germany; i Department of Pediatrics, University Hospital Wuerzburg, Wuerzburg, Germany; j Germany Translational Lung Research Center (TLRC) and German Center for Lung Research (DZL), Heidelberg, Germany

**Keywords:** Antibiotics, preterm infants, vaccination, microbiome, sex-differences

## Abstract

Neonatal sepsis represents a major risk in preterm infant care, resulting in widespread early-life antibiotic exposure. While the latter has been linked to immune maturation in term-born neonates, its impact on preterm immune development remains unclear. The aim of this prospective observational study was to investigate the effect of early antibiotic exposure on vaccine titers at a corrected age of four months. To achieve this, blood and stool samples were analyzed from 69 preterm infants (<32 weeks gestational age; 35 with 34 without antibiotic exposure during the first postnatal week) at postnatal day 14 and again at four months corrected age. We assessed vaccine-induced antibody titers against *Bordetella pertussis* and *Haemophilus influenza*e, immune cell profiles (flow cytometry), gut microbiome composition (16S rRNA sequencing), and plasma amino acid and acylcarnitine levels (tandem mass spectrometry). Preterm infants exposed to early antibiotics showed reduced antibody titers following vaccination, with differences appearing more pronounced in girls. Antibiotic-exposed girls displayed increased monocytes and myeloid-derived suppressor cells (MDSCs), both of which inversely correlated with antibody titers. Early antibiotic exposure was associated with differences in microbial community types at postnatal day 14, with *Klebsiella*-dominated and *Bifidobacteria*-lacking communities occurring more frequently in antibiotic-exposed infants. Antibiotic-exposed girls exhibited distinct metabolomic alterations, including elevated levels of two unsaturated fatty acids that negatively correlated with monocyte and MDSC abundance. Our findings suggest that early antibiotic exposure impairs vaccine responses in preterm infants and indicates a potentially sex-specific susceptibility. Antibiotic-driven changes in the microbiome and metabolome may sustain suppressive innate immune cell populations, which may in turn weaken adaptive responses to vaccination.

## Introduction

Bacterial infections are among the leading causes of death in preterm infants.^1^ Approximately one in three very low birthweight infants (VLBW < 1500 g) develops at least one bacterial infection during their stay in the neonatal intensive care unit (NICU).^1^ The incidence of bacterial infections in preterm infants increases markedly with decreasing gestational age.^2^ In addition to high mortality rates, neonatal bacterial infections can lead to long-term and often serious consequences for the affected infant, such as chronic lung disease or impaired neurologic development.^1^ ^,^ ^3^ For these reasons, up to 80% or more of all preterm infants receive antibiotics during their first week of life.^4^ ^,^ ^5^ However, recent studies have demonstrated that perinatal antibiotic exposure itself is associated with an increased risk of infections or necrotizing enterocolitis (NEC) in the neonatal period^4^ ^,^ ^6^ ^,^ ^7^ and contributes to chronic health problems, such as allergy, asthma, and obesity later in life.^8-10^ These adverse effects of early antibiotic exposure are mainly attributed to alterations in the intestinal microbiome and its interaction with the developing immune system.^11-14^

Vaccination saves more than 2.5 million lives each year. However, about 3%–13% of all infants remain unprotected against vaccine-preventable diseases due to limited vaccine effectiveness, both in low- and high-income countries.^15^ ^,^ ^16^ Recent studies have shown that the composition of the intestinal microbiome correlates with vaccine responses to certain antigens.^17^ ^,^ ^18^ In addition, germ-free as well as antibiotic-exposed mice exhibit reduced vaccine responses, especially when treated early in life.^19^ ^,^ ^20^ A recent study showed that exposure to neonatal, but not intrapartum, antibiotics resulted in significantly lower antibody titers against various vaccines in healthy term newborns and coincided with a reduced abundance of *Bifidobacterium* species at the time of vaccination.^21^ These data indicate that early antibiotic exposure exerts profound effects on immune development during the neonatal period. However, similar data for preterm infants are lacking.

In this study, we assessed whether early antibiotic exposure affects vaccine responses in preterm infants at a corrected age of four months. We prospectively recruited infants born preterm without or with antibiotic exposure during their first week of life and analyzed vaccine titers, immune profiles, microbiome patterns, and metabolite profiles before and after the first three vaccinations with a polyvalent vaccine containing *Hemophilus influenzae* and *Bordetella pertussis* to better understand the mechanisms underlying the antibiotic-driven dysregulation of immune responses during early life.

## Methods

### Study design and patients

We performed a prospective observational study in preterm infants born between 24 0/7 and 31 6/7 weeks` gestation. The main outcome parameter was antibody titers *H. influenzae* type B and *B. pertussis* at a corrected age of four months. Additional outcomes included immune cell composition, microbiome composition, and levels of selected serum metabolites at 14 d of age and at a corrected age of four months. The study flow is shown in Supplementary Figure 1A. The study was approved by the Ethics Committee of the Medical Faculty at the University of Tübingen (Project number: 368/2019BO1 and 024/2022B01) and registered at ClinicalTrials.gov under registration number NCT05563753. Preterm infants born between 24 0/7 and 31 6/7 weeks` gestation at Tübingen University Hospital who received at least one dose of antibiotics within the first week of life, and preterm infants who did not receive antibiotics in the first week of life, were enrolled between January 2020 and December 2024 at the Department of Neonatology at Tübingen University Hospital. Due to the standard clinical practice in our department, where laboratory parameters are reassessed after 48 h following the initiation of antibiotic therapy, and further treatment decisions are then made based on these results, all infants in the antibiotic group received antibiotics for at least 48 h. Exclusion criteria were genetic disorders, chronic infections, hematological disorders, immunoglobulin administration within the first 60 d of life, immunodeficiencies, and maternal hepatitis B infection. No mothers of a preterm infant in the analyzed cohort received immunosuppressive treatment.

According to the standard clinical practice in our department, all preterm infants included in the study received the probiotic combination preparation Infloran®, consisting of 1 billion CFU *Bifidobacterium bifidum* and 1 billion CFU *Lactobacillus acidophilus,* once daily from the first day of life until completion of 32 weeks of gestational age or until six weeks of age, whichever occurred later.

### Clinical data

Demographic, clinical, and nutritional data were collected from our institutional electronic hospital records (Neodat for demographic and clinical data and Medipaed for nutritional data, both from PaedSoft Software, Tübingen).

*Gestational age at birth (GA)* was calculated from the best obstetric estimate based on early prenatal ultrasound and obstetric examination and provided as postmenstrual age (PMA).

*Small for gestational age (SGA)* was defined as birth weight < 10th percentile according to GA.^22^

*Prenatal antibiotic therapy* was defined as maternal antibiotic exposure within the 14 d preceding delivery. Intrapartum antibiotic administration was not included in this definition. Prenatally administered antibiotics to the mother included cefuroxime (*n* = 25), ampicillin (*n* = 3), piperacillin/tazobactam (*n* = 4), clarithromycin (*n* = 2), gentamicin (*n* = 2), and vancomycin (*n* = 1).

*Postnatal maternal antibiotic therapy* was defined as maternal antibiotic exposure during the hospital stay of the infant. Postnatally administered antibiotics to the mother included cefuroxime (*n* = 27), piperacillin/tazobactam (*n* = 1), and clindamycin (*n* = 2).

*The proportion of breast milk feeding* was assessed as the proportion of breast milk in the total nutritional intake at day 14 and at delivery.

### Vaccine schedule

Preterm infants were vaccinated with the combination 6-in-1 vaccine (Infanrix Hexa, GSK) and with PCV13 (Prevenar 13, Pfizer) and at 8 weeks and 3 and 4 months in accordance with the recommendations of the German Standing Committee on Vaccination (STIKO). Infanrix Hexa contains diphtheria (≥30IU) and tetanus toxoids (≥40IU), acellular *B. pertussis* antigens (25 µg pertussis toxoid, 25  µg filamentous hemagglutinin and 8 µg pertactin), hepatitis B surface antigen (HBs, 10 µg), inactivated poliovirus types 1–3 (40, 8 and 32 D-antigen units) and *H. influenzae* type b polysaccharide (10 µg) conjugated to tetanus toxoid per 0.5 mL dose. Vaccinations were administered according to the chronological age of the infants. All vaccines were administered intramuscularly.

### Sample collection

Stool samples were collected at postnatal day 14 by the attending nurse and at the corrected age of four months by a parent at home. A spoonful of fecal material was collected in DNA/RNA Shield Fecal Collection tubes (Zymo Research), and samples were directly stored at −80 °C (day 14 samples) or sent by participants' parents to the study center by mail and then stored at −80 °C until further processing.

Whole blood stabilized with Ethylenediaminetetraacetic acid (EDTA, for immune cell profiling and metabolite analyses) and native blood samples (for analysis of vaccination titers) were collected at both timepoints by the attending physician. Immediately upon blood taking, peripheral blood mononuclear cells (PBMC) were isolated from EDTA blood and then stored in freezing media (RPMI + 20% FCS + 20% DMSO) at −80 °C until further processing. The native blood samples were processed by the Institute of Microbiology and Hygiene at Tübingen University Hospital. Antibody titers against the pathogens *H. influenzae* type B (*H. influenzae* B IgG ELISA, IBL International, Hamburg, Germany) and *B. pertussis* were determined from the serum by ELISA. For *B. pertussis* antibody titers (IgG) against the *B. pertussis* toxin (PT- IgA-ELISA, Euroimmun, Lübeck, Germany) and the surface protein filamentous hemagglutinin (FHA) (FHA-IgG-ELISA, Euroimmun, Lübeck, Germany) were determined. Due to the limited blood volume that can safely be obtained from very preterm infants, particularly at the early sampling time point on postnatal day 14, it was not feasible to assess antibody responses to all vaccine antigens. Therefore, antibody titers were measured for selected antigens only (protein and polysaccharide antigens) to represent different types of vaccine-induced immune responses. Supplementary Figure 1B shows the number of samples that were available from study participants at the two timepoints.

### Cell isolation and flow cytometry

Human peripheral blood mononuclear cells (PBMC) were prepared from EDTA blood samples by density gradient centrifugation (Pancoll lymphocyte separation medium, PanBiotech, Aidenbach, Germany). For quantification of immune cell subsets from PBMC, cells were pre-gated to living CD45-expressing leukocytes. Supplementary Figure 2 shows the gating strategy for the different immune cell types. Antibodies used for extracellular staining of human cells were purchased from BioLegend, San Diego, USA (CD45 APC-Cy7 (clone 2D1, Cat#: 368516), CD3 PE-Cy7 (clone HIT3a, Cat#: 300316), CD4 PerCp (clone RPA-T4, Cat#: 300527), CCR6 APC (clone G034E3; Cat#: 353415), CD25 BV421 (clone M-A251, Cat#: 356114), CCR7 PE (clone G043H7, Cat#: 353203), CD45 RO PerCp (clone UCHL1, Cat#: 304221), CD8 APC-Cy7 (clone SK1, Cat#: 344713), CD4 Pacific Blue (clone RPA-T4, Cat#: 300524), CD19 APC (clone HIB19, Cat#: 302212), CD38 PE (clone S170157, Cat#: 397203), IgD PerCp (clone IA6-2, Cat#: 348233), CD27 PE-Cy7 (clone S20020G, Cat#: 384911), CD10 APC-Cy7 (clone HI10a, Cat#: 312212), CD45 Pacific Blue (clone HI30, Cat#: 304022), CD138 FITC (clone MI15, Cat#: 356507), IgM APC-Cy7 (clone MHM-88, Cat#: 314519), CD14 PE-Cy7 (clone M5E2, Cat#: 301813)), R&D, Minneapolis, USA (CCR4 FITC (clone 205410, Cat#: FAB1567F), BD Bioscience, Heidelberg, Germany (CXCR3 PE (clone 1C6, Cat#: 560928)) and Miltenyi, Bergisch-Gladbach, Germany (CD45 PE (clone 5B1, Cat#: 130-110-632), CD66b APC (clone G10F5, Cat#: 130-117-692)).

Data acquisition was performed with a FACSCanto II and analyzed via FlowJo V10 (FlowJo, LLC, Ashland, Oregon, USA).

### DNA extraction and 16 s rRNA sequencing

Genomic DNA from stool samples was extracted using the ZymoBIOMICS DNA/RNA Miniprep Kit (Zymo Research) according to manufacturer's instructions. In brief, samples were bead-beated using ZR BashingBead™ Lysis Tubes, and the DNA was purified via spin-column and filtered to remove PCR inhibitors. Purified DNA was quantified with a Qubit fluorometer using the Invitrogen Qubit DNA Broad Range (BR) Assay KitsExtracted DNA was stored at −80 °C for preservation before further PCR amplification and sequencing.

16 s rRNA sequencing was performed by the Core Facility Genomics, Medical Faculty, University Hospital Tübingen/DFG-funded NGS Competence Center NCCT Tübingen (INST 37/1049-1) as described in refs. 23 and 24 Genomic DNA was normalized to 50 ng input for library preparation. The first step PCR was performed in 15 µL reactions including KAPA HiFi HotStart ReadyMix (Roche), 515F and 806 R (~350 bp fragment of the 16S V4 region)^13^ and template DNA (PCR program: 95 °C for 3 min, 28× (98 °C for 20 s, 55 °C for 15 s, 72 °C for 15 s), 72 °C for 5 min). First, PCR products were purified using 12 µL AMPure XP beads and eluted in 26 µL 10 mM Tris–HCl. Indexing was performed in the second PCR, including KAPA HiFi HotStart ReadyMix (Roche), index primer mix (Illumina DNA UD Index Sets), and purified first PCR product as template (PCR program: 95 °C for 3 min, 8x (95 °C for 30 s, 55 °C for 30 s, 72 °C for 30 s), 72 °C for 5 min). After another bead purification (14 µL AMPure XP beads, eluted in 15 µL 10 mM Tris–HCl) the libraries were checked for correct fragment length on an agarose gel, quantified with a Qubit dsDNA BR Assay Kit (Thermo Fisher) and pooled equimolarly. The pool was sequenced on an Illumina MiSeq device with a v2 sequencing kit with 2 × 250 bp read length and 20% PhiX spike-in. An average depth of 150k reads per sample was achieved.

### Bioinformatic analysis

#### Demultiplexing and quality control

Data was processed using nf-core/demultiplex (version 1.4.2)^25^ of the nf-core collection of workflows, using reproducible software environments from the Bioconda^26^ and Biocontainers^27^ projects. Read quality was thereby checked with fastp (version 0.23.4).^28^ The pipeline was executed with Nextflow (version 23.10.1).^29^

#### 16S amplicon analysis

16S amplicon analysis was performed as described in ref. 30 Data was processed using nf-core/ampliseq (version 2.9.0)^31^ of the nf-core collection of workflows,^25^ using reproducible software environments from the Bioconda^26^ and Biocontainers^27^ projects. Data quality was evaluated with FastQC (version 0.12.1) and summarized with MultiQC (version 1.21).^32^ Cutadapt (version 4.6)^33^ trimmed primers and all untrimmed sequences were discarded. Sequences that did not contain primer sequences were considered artifacts. Adapter and primer-free sequences were processed sample-wise (independent) with DADA2 (version 1.30.0)^34^ to eliminate PhiX contamination, trim reads (before median quality drops below 25 and at least 75% of reads are retained), discard reads with >2 expected errors, correct errors, merge read pairs, and remove polymerase chain reaction (PCR) primers. Taxonomic classification was performed by DADA2 and the database “Silva 138.1 prokaryotic SSU.”^35^ ASV sequences, abundance and DADA2 taxonomic assignments were loaded into QIIME2 (version 2023.7.0).^36^

### Tandem mass spectrometry (MS/MS)

Due to limited serum volumes, a targeted metabolomics approach focusing on amino acids and acylcarnitines was used. These metabolite classes are closely linked to immune cell metabolism and mitochondrial energy pathways and are partly influenced by microbial metabolism in the gut.^37^ ^,^ ^38^ MS/MS analyses of plasma samples to measure amino acids and acylcarnitines were performed by the Dietmar-Hopp-Stoffwechselzentrum Heidelberg as described in ref.^39^ For analyses of amino acids and acylcarnitines in plasma samples, 4.7 mm punches were taken from a blank filter card (Whatman 903 paper) and placed into a 96-well filter plate. A volume of 5 µL of each plasma sample was pipetted onto a punch and dried overnight at room temperature. Sample preparation was carried out using the reagents of the MassChrom® kit for the analysis of amino acids and acylcarnitines from dried blood for newborn screening (57000F, non-derivatized, Chromsystems Instrument and Chemicals GmbH, Gräfelfing, Germany). The following steps were performed: 150 µL of a dilution of internal standards (internal standard—succinylacetone:internal standard, 1:1, v/v) and 75 µL of the extraction buffer—succinylacetone were added to the punches. Analytes were extracted by incubating for 30 min at 45 °C and 600 rpm on a thermoshaker (Biosan, Riga, Latvia). After centrifugation at 3200 g for 2 min into a 96-well V-bottom plate, the plate was incubated for 20 min at room temperature. For the measurement, 10 µL of the supernatant was injected into the MS/MS system via flow injection (FIA-MS/MS). Amino acids and acylcarnitines were quantified using electrospray ionization tandem mass spectrometry (ESI-MS/MS) with a Waters Xevo TQD triple quadrupole mass spectrometer (Waters GmbH, Eschborn, Germany), equipped with an electrospray ion source and MassLynx software.

### Statistical analysis

All statistical analyses were performed using Python (version 3.11). The following Python libraries were used: Matplotlib (version 3.10.0), Numpy (version 2.2.2), Openpyxl (version 3.1.5), Pandas (version 2.2.3), Scikit-bio (version 0.6.3), Scikit-learn (version 1.6.1), Scikit-posthocs (version 0.11.4), Scipy (version 1.15.1), Seaborn (version 0.13.2), Statannotations (version 0.7.1), Statsmodels (version 0.14.4). Differential abundance analysis was performed using ANCOM-BC as implemented in the “ANCOMBC” package in R (version 4.5.2).

#### Clinical data

To describe clinical characteristics of infants without and with antibiotic exposure in their first postnatal week, categorical variables were summarized by absolute and relative frequencies, and numerical variables were assessed for normality using the Shapiro–Wilk test and summarized with mean and standard deviation (for normally distributed data) or median and interquartile range (for non-normally distributed data).

For group comparisons, categorical variables were analyzed using the chi-squared test or Fisher's exact test. Numerical variables were compared using Student's *t*-test (for normally distributed data) or Mann–Whitney *U* test (for non-normal data). Multiple testing was done using the Benjamini–Hochberg procedure to control for any false discovery rates. An adjusted *p*-value of <0.05 was considered statistically significant.

#### Vaccine titers

Antibody titers were log-transformed prior to statistical analysis to account for their skewed distribution. For visualization, scatter plots are shown with geometric means and 95% confidence intervals on a logarithmic scale. Differences between groups were assessed by *t*-test. All tests were performed two-tailed. A correction for multiple testing was performed using Benjamini–Hochberg procedure. The figures were created using Graphpad Prism 10.1.1. For each antibody titer at a corrected age of four months, multiple linear regression models were fitted. The dependent variable was the antibody titer, while independent variables included sex, gestational age, birth weight, antibiotic exposure in the first week of life, late postnatal antibiotic exposure, age [days] at the second vaccination, and the time interval between vaccination and antibody measurement [days]. The independent variables were selected based on univariable comparisons of the clinical characteristics between the two groups. Since biological and clinical relationships are often multicausal and effects can change in multivariable models, a more liberal threshold of *p* < 0.1 was chosen in the univariable analysis to also include trends in the linear regression model. Sex was added as an independent variable based on evidence from the literature showing sex-specific differences in vaccine titers.^40^ ^,^ ^41^ To assess whether the association between early antibiotic exposure and vaccine titers differed by sex, an interaction term between early antibiotic exposure and sex was tested in each model. For visualization, sex-stratified model-based interaction plots were generated for each vaccine titer.

#### Immune cell analyses

Proportions of immune cells in PBMC were assessed for normality using the Shapiro–Wilk test, and differences between groups were assessed by ANOVA with Šídák's multiple comparisons test or Kruskal–Wallis test with Dunn's multiple comparisons test. Post-hoc tests were only calculated for the group comparisons annotated in the figures. The figures were created using Graphpad Prism 10.1.1. To account for repeated measurements within individuals, immune cell populations were analyzed using linear mixed-effects models (LMM) with patient ID as a random effect and timepoint, early antibiotic exposure, gestational age, and sex as fixed effects. A timepoint × early antibiotic exposure interaction term was included to assess differences in temporal dynamics between groups. *P-values* were adjusted for multiple testing using the Benjamini–Hochberg false discovery rate method. Pairwise correlations between adaptive and innate immune cell subsets and vaccine titers were assessed using Spearman's rank correlation coefficient (*ρ*), as normality of the data could not be assumed. For each cell type–titer pair, raw *p-values* were calculated and subsequently adjusted for multiple testing using the Benjamini–Hochberg procedure to control the false discovery rate (FDR) separately for adaptive and innate immune cells. As a sensitivity analysis, linear regression models were additionally fitted to adjust for potential confounders, including gestational age and sex.

#### Microbiome analyses

To examine differences in alpha diversity between groups, the normality of the distributions was tested using the Shapiro–Wilk test. If all groups were normally distributed, one-way ANOVA was performed, followed by Šídák's post-hoc test for pairwise comparisons. If normality assumptions were violated, the Kruskal–Wallis test was applied, followed by Dunn's post-hoc test. Post-hoc tests were only calculated for group comparisons annotated in the figures. Alpha diversities were visualized using Graphpad Prism 10.1.1. Associations between antibiotic exposure and alpha diversity were further assessed using LMMs. Models were adjusted for gestational age, sex, and delivery mode, which are known factors influencing the neonatal gut microbiome. To explore potential sex-specific effects, additional models including an interaction term between antibiotic exposure and sex were fitted. For analysis of beta diversity, a Bray–Curtis dissimilarity matrix was computed using the Pdist function from the Scipy.spatial.distance package and converted into a square matrix with Squareform. The Bray-Curtis dissimilarity matrix served as input for principal coordinates analysis (PCoA) implemented in scikit-bio. The first two principal coordinate axes were used to visualize differences in microbial community composition. To identify groups of similar samples, hierarchical agglomerative clustering with Ward's linkage method was applied to all PCoA coordinates. The optimal number of clusters was chosen based on the inspection of the resulting dendrogram. To visualize the temporal dynamics of community types and the impact of antibiotic exposure or sex on community types, stacked bar plots were generated showing the relative proportion of each cluster at the two sampling time points (T1 and 2). Differential abundance analysis between clusters was performed using ANCOM-BC (Analysis of Composition of Microbiomes with Bias Correction). The analysis was conducted on genus-level abundance counts, and cluster assignment was used as the grouping variable. *p*-values were adjusted for multiple testing using the Benjamini–Hochberg false discovery rate (FDR) correction, and genera with adjusted *p*-values < 0.05 were considered significantly differentially abundant. To investigate temporal stability, cluster assignments defined at T1 were mapped to the corresponding samples collected at T2, and differences in genus-level abundances were again assessed using ANCOM-BC. The taxonomic composition of the clusters at both timepoints was visualized using stacked bar plots. To evaluate whether cluster membership was associated with antibiotic exposure or sex, contingency tables of cluster counts by group were constructed and tested using the chi^2^ test. To investigate associations between gut microbiota composition at T1 and vaccine-specific antibody responses, a multi-output Random Forest regressor was trained. Bacterial genera abundances were used as predictors (X), while vaccine antibody titers served as response variables (Y). To assess the robustness of microbial predictors with respect to potential clinical confounding, additional models were trained, including gestational age, sex, and delivery mode as additional predictor variables. The dataset was split into training and test sets (80/20). Both predictors and response variables were standardized using z-score normalization. A multi-output regression approach was applied using Random Forest Regression. Hyperparameters of the base Random Forest were optimized via grid search with 3-fold cross-validation. The parameters were tuned as follows: number of trees *100, 200*, maximum tree depth *none, 10, 20*, maximum number of samples required to split a node *2, 5*. The model with the best cross-validated *R*² score was selected and subsequently retrained on the full training dataset. Predictions were obtained for the test set, and performance was evaluated using the coefficient of determination (*R*²) and the mean squared error (MSE). To account for variability due to data partitioning, a stability analysis was performed. The modeling procedure was repeated 50 times with different random splits of the data (random states 0–49), each time retraining the optimized Random Forest model. For each run, feature importances were calculated, and the frequency with which individual genus appeared among the top 10 predictors was recorded. Mean and standard deviation of importances across runs were computed, and the most stable predictors were visualized in a heatmap.

#### Metabolite analyses

To explore patterns in the metabolite profiles of preterm infants, Principal Component Analysis (PCA) was performed. Prior to PCA, metabolites with more than 50% missing or zero values were excluded to reduce noise. The PCA was conducted on the remaining metabolite data, and the first two principal components (PC1 and PC2) were extracted for visualization. To statistically assess differences in metabolite profiles between timepoints, a Permutational Multivariate Analysis of Variance (PERMANOVA) was performed. In this study, PERMANOVA was applied to Bray-Curtis distances calculated from the filtered metabolite data (after exclusion of metabolites with more than 50% missing or zero values, as well as features with very low variance (<10^−5^ )). Statistical significance was determined using 999 permutations. To identify associations between metabolite levels and innate immune cells (monocytes and MDSC) at a corrected age of four months (T2), pairwise associations between each metabolite from the filtered metabolite data and each immune cell subset were assessed using Spearman's rank correlation. Significant correlations were visualized in a heatmap. For testing for differences in metabolite levels between male and female infants exposed to early antibiotics, a Shapiro–Wilk test was performed to test for normal distribution. An unpaired *t*-test was used for comparisons between normally distributed data, and a Mann–Whitney test was used for comparisons between non-normally distributed data. A correction for multiple testing was performed using the Benjamini–Hochberg procedure. An adjusted *p*-value of <0.05 was considered statistically significant. The figures were created using Graphpad Prism 10.1.1.

## Results

### Study population

A total of 95 patients were included in the study, but 26 had to be excluded from the final analysis because they had not received three vaccinations at the time of the follow-up at four months. A total of 69 preterm infants were thus included in the final analysis. Of these, 34 (49.3%) were exposed to antibiotics in their first postnatal week (ABT group), and 35 (50.7%) were not (no ABT group) (Supplementary Figure 1). Only two of the 34 infants in the ABT group had proven sepsis, one with proven early-onset (EOS) and one with late-onset sepsis (LOS) on postnatal day 6; both had *S. aureus*–positive blood cultures. Median duration of antibiotic therapy with start in the first week of life was 3.5 d (IQR 2 d). Of the 34 infants in the ABT group, 29 (85%) received ampicillin and tobramycin, two (6%) additionally received azithromycin, and one (3%) received cefotaxime. Three infants (9%) received piperacillin/tazobactam, while two (6%) received a combination of ampicillin, amikacin, and cefotaxime. Patients in the ABT group had a slightly lower gestational age and lower birth weight and were more likely to receive antibiotics after the first week of life. After correction for multiple testing, none of these group differences remained significant. Table 1 shows the basic perinatal and clinical patient characteristics for both groups.

**Table 1.** Patient characteristics of patients without (No ABT) and with (ABT) antibiotic exposure in their first postnatal week.

	No ABT (*n* = 35)	ABT (*n* = 34)	*p*-value	adj. *p*-value
Gestational age [median (IQR)]	30.1 (1.9)	29.5 (2.0)	0.07	0.19
Birth weight (g) [mean (SD)]	1320 (320)	1160 (400)	0.08	0.19
Sex (male)	18 (51.4)	14 (41.2)	0.47	0.69
APGAR 10 [median (IQR)]	10 (1)	9 (1)	0.18	0.28
Arterial cord blood pH [median (IQR)]	7.3 (0.1)	7.3 (0.1)	0.33	0.42
Arterial cord blood BE [median (IQR)]	−2.1 (3.7)	−1.7 (3.0)	0.47	0.51
Mode of delivery				
Vaginally	3 (8.6)	2 (5.9)	0.41	0.69
Elective C/S	16 (45.7)	11 (32.4)		
Emergency C/S	16 (45.7)	21 (61.8)		
Prenatal antibiotics (*n* = 67)^a^	*n* = 34 13 (38.2)	*n* = 33 13 (39.4)	0.83	1.0
Duration [median (IQR)]	0 (5.5)	0 (3.8)	0.90	0.90
Postnatal maternal antibiotics	15 (42.9)	17 (50)	0.63	0.87
Duration [median (IQR)]	0 (2)	1 (3)	0.25	0.36
Late postnatal antibiotics	2 (5.7)	9 (26.5)	**0.02**	0.25
Multiples	21 (60.0)	16 (47.1)	0.33	0.64
SGA	3 (8.6)	7 (20.6)	0.19	0.44
AIS	3 (8.6)	4 (11.8)	0.71	0.87
pPROM (*n* = 68)	*n* = 34 7 (20.5)	13 (38.2)	0.18	0.44
Preeclampsia	2 (5.7)	5 (14.7)	0.26	0.55
Mechanical ventilation	6 (17.1)	12 (35.3)	0.11	0.43
Proportion of breast milk feeding at day 14 (%) [median (IQR)]	50 (41.5)	50 (68.8)	0.45	0.51
Proportion of breast milk feeding at delivery (%) [median (IQR)]	50 (39.5)	58.5 (78.8)	0.68	0.68
Age at first immunization (days) [median (IQR)]	65 (14)	60 (5)	0.21	0.30
Age at second immunization (days) [median (IQR)]	104.5 (23.3)	93 (19)	0.05	0.18
Age at third immunization (days) [median (IQR)]	147 (23)	137 (25.5)	0.17	0.28
Time interval between 3rd immunization and titer assessment (days) [median (IQR)]	50 (28)	53 (28.8)	0.06	0.19

IQR, interquartile range; SGA, small for gestational age; IAI, intraamniotic infection; pPROM, preterm premature rupture of the membranes; APGAR 10, APGAR score at 10 min after birth.

Bold values indicate statistical significance (*p* < 0.05).

^a^ In the last 14 d before pregnancy, intrapartum antibiotic prophylaxis was not considered.

### Decreased vaccine titers in preterm infants with antibiotic exposure in the first postnatal week

**Figure 1.**
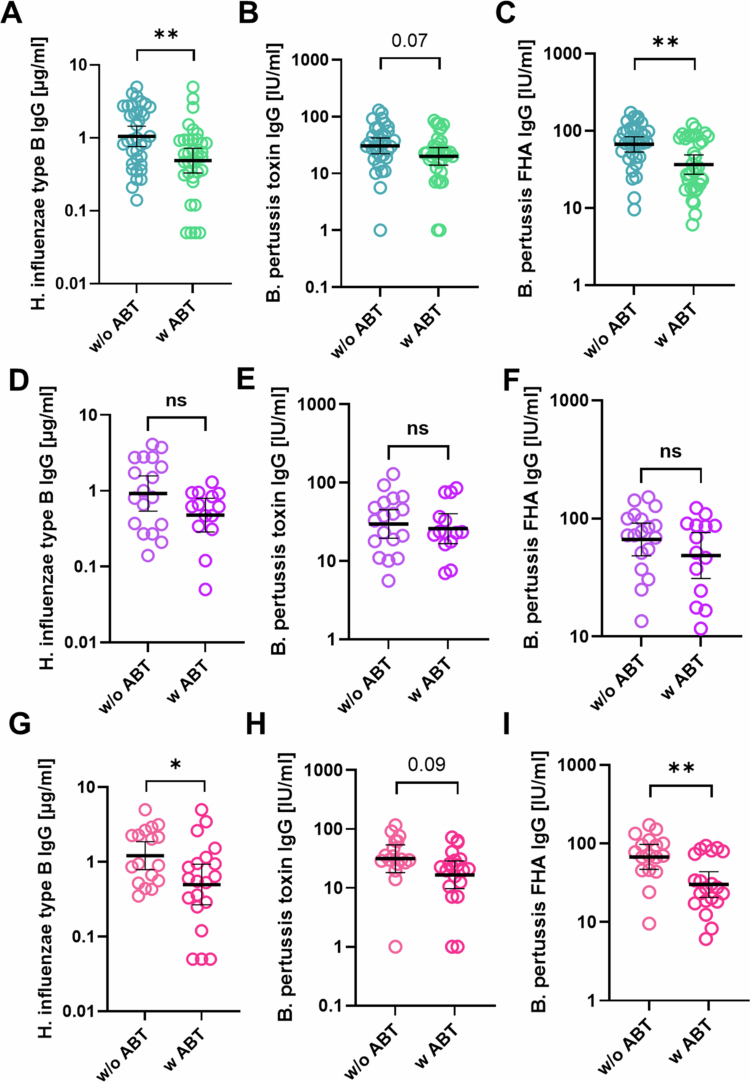
Vaccination titers against *H. influenzae* type B and *B. pertussis* in preterm infants without and with early antibiotic exposure. Vaccination titers against *H. influenzae* type B and *B. pertussis* (*B. pertussis* toxin IgG and *B. pertussis* FHA IgG) were determined from native blood samples collected at a corrected age of four months. The scatter plots with geometric means and 95% confidence intervals show vaccination titers against *H. influenzae* type B (A + D + G), *B. pertussis* toxin (B + E + H) and *B. pertussis* FHA (C + F + I) in preterm infants without (w/o ABT, *n* = 35) and with (w ABT, *n* = 34) antibiotic exposure in their first postnatal week. Green plots (A–C) show the whole cohort, violet plots (D–F) show only male infants (w/o ABT *n* = 18, W ABT *n* = 14) and pink plots (G–I) show only female infants (w/o ABT *n* = 17 and w ABT *n* = 19). **p < 0.01, *p < 0.05, ns not significant, *t*-test (after log-transformation of vaccine titers) and correction for multiple testing using the Benjamini–Hochberg procedure.

Analysis of vaccine titers against *H. influenzae* type B and *B. pertussis* at a corrected age of 4 months showed significantly lower IgG levels against *H. influenzae* type B and *B. pertussis* FHA in preterm infants with antibiotics in the first postnatal week compared to those without (Figure 1A–C). Differences also remained significant when infants who had culture-positive sepsis were excluded from the early antibiotic exposure group (Supplementary Figure 3A–C). Among infants with early antibiotic exposure, 6/34 (18%), 1/34 (3%), and 0/34 (0%) had antibody titers below the protective thresholds reported in the literature^21^ for *H. influenzae* type B (150 ng/ml), *B. pertussis* toxin (5000 mIU/ml), and *B. pertussis* FHA (5000 mIU/ml) respectively, compared with 1/35 (3%), 1/35 (3%), and 0/35 (0%) in infants without early antibiotic exposure (adj. *p*-values *p* = 0.05, *p* = 1 and *p* = 1 in Fisher's exact test). In a multivariate regression, adjusting for variables that differed between groups with an uncorrected *p*-value of <0.1 in univariate analysis and the variable sex (based on evidence from the literature showing sex-specific differences in vaccine titers,^40^ ^,^ ^41^ early antibiotic exposure remained significantly associated with lower vaccine titers. In addition, our regression model showed an association between vaccine titers for *B. pertussis* FHA and the timing of the second vaccination and for *B. pertussis* toxin and the time interval between the last vaccination and antibody assessment (Table 2).

**Table 2.** Variables associated with vaccination titer levels in multivariate regression.

variable	*p*-value (*H. influenzae* type B IgG)	*p*-value (*B. pertussis* toxin IgG)	*p*-value (*B. pertussis* FHA IgG)
Sex	0.51	0.79	0.24
Gestational age	0.91	0.07	1
Birth weight (g)	0.94	0.10	0.47
Early antibiotic exposure	**0.01**	0.09	**<0.01**
Late postnatal antibiotics	0.62	0.31	0.62
Age at 2. immunization	0.80	0.64	**0.03**
Time interval between vaccination and antibody assessment	0.19	**0.01**	0.57

Bold values indicate statistical significance (*p* < 0.05).

Exploratory sex-stratified analyses suggested a stronger reduction in vaccine titers in girls exposed to early antibiotics, whereas only a non-significant trend was observed in boys (Figure 1D–I). Interaction analyses between sex and antibiotic exposure revealed a significant interaction of antibiotic exposure and sex for *B. pertussis* FHA (adjusted *p*-value for interaction 0.01), but not for *B. pertussis* toxin (adjusted *p*-value for interaction 0.16) or *H. influenzae* type B (adjusted *p*-value for interaction 0.56). Supplementary Figure 4A–C shows model-based interaction plots with predicted antibody titers according to antibiotic exposure and sex, adjusted for gestational age, birth weight, antibiotic exposure after the first week of life, timing of vaccination, and the interval between vaccination and blood sampling.

At 14 d of age, i.e., before the first vaccination, there were no differences in antibody titers (maternal passive immunity) either in the entire cohort or in the sex-specific analysis, except for HiB in the cohort of male preterm infants, in which infants exposed to early antibiotics had lower antibody titers (Supplementary Figure 3D–L).

### Persistence of myeloid cells in girls following early antibiotic exposure

In order to identify a possible link between reduced vaccine titers following early antibiotic exposure and altered adaptive immune development, flow cytometric analysis of B and T cells and their subpopulations was performed. Infants with early antibiotic exposure had more T helper cells and less cytotoxic T cells at T2. Beyond that, there were no significant differences in B and T cells or their subpopulations between infants with and without early antibiotic exposure at any time point (Supplementary Figure 4A–X). Changes in T and B cell populations over time and the effect of early antibiotic exposure were further analyzed using linear mixed models (LMM), adjusting for gestational age and sex. Several immune cell populations (T cells, T helper 2 cells, regulatory T cells, central memory T helper cells, naïve T helper cells, effector T helper cells, central memory cytotoxic T cells, effector cytotoxic T cells, plasmablasts, class-switched B cells, naïve B cells) showed significant changes over time (Supplementary table, whereas early antibiotic exposure did not significantly influence their temporal dynamics (no significant time × antibiotic exposure interaction).

Correlation analyses between B and T cells and their subtypes and the three measured vaccine titers also showed no significant correlations (data not shown).

**Figure 2.**
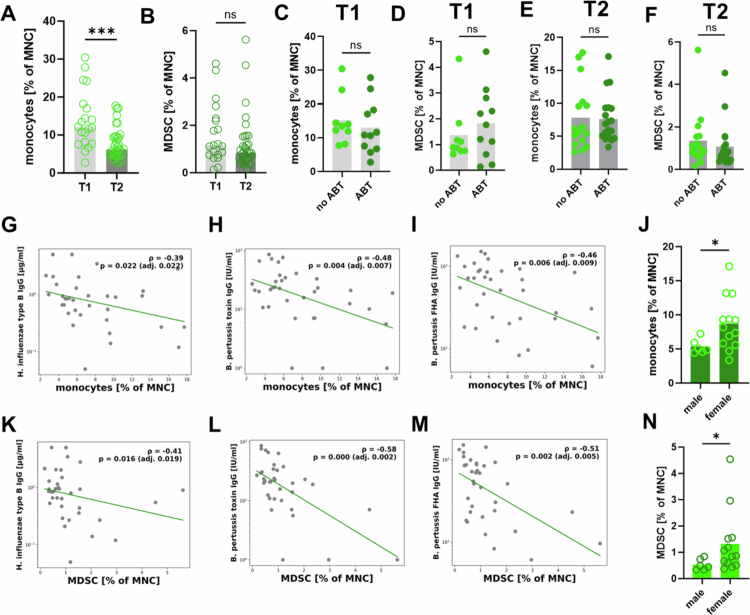
Innate immune cell subsets and their correlations with vaccine titers. Mononuclear cells (MNC) were purified from the peripheral blood of preterm infants on postnatal day 14 (*n* = 20) and at a corrected age of 4 months (*n* = 35), and the percentage of monocytes and myeloid-derived suppressor cells (MDSC) in MNC was determined by flow cytometry. (A + B) The bar charts show the proportion of monocytes (A) and MDSC (B) in all MNCs on postnatal day 14 (light grey bars) and at a corrected age of four months (dark grey bars). (C–F) The bar charts show the proportion of monocytes (C + E) and MDSC (D + F) in all MNC on postnatal day 14 (C + D) and at a corrected age of four months (E + F), depending on whether they were exposed to early antibiotic therapy. ***p < 0.001, ns not significant; t-test with correction for multiple testing using the Benjamini–Hochberg procedure. (G-I, K-M) Correlation analyses were performed for monocytes and MDSC with the vaccine titers for *B. pertussis* toxin, *B. pertussis* FHA, and H. influenzae type B. Correlation plots show the correlation of monocytes (G–I) and MDSC (K–M) with the three vaccine titers (after log-transformation) as assessed by Spearman correlation. (J + N) The bar charts show the proportion of monocytes (J) and MDSC (N) in all MNCs from male and female antibiotic-exposed infants at a corrected age of four months. *p < 0.05, Mann–Whitney test.

Regarding myeloid cells, there was a decrease in the proportion of monocytes from T1 to T2 (Figure 2A, adj. *p*-value 0.02 in the LMM), while MDSC decreased only slightly (Figure 2B, adj *p*-value 0.12 in the LMM). Early antibiotic exposure had no effect on the proportion of the two cell types at either T1 or T2 (Figure 2C–F). Correlation analyses between monocytes and MDSC and the three measured vaccine titers showed no significant correlations on postnatal day 14 (data not shown) but negative correlations for both innate immune cell types with all vaccine titers at a corrected age of four months (adjusted *p*-values 0.022, 0.007 and 0.009 for correlations of monocytes with *H. influenzae* type B IgG, *B. pertussis* toxin IgG and *B. pertussis* FHA IgG and 0.016, 0.002 and 0.005 for MDSC with *H. influenzae* type B IgG, *B. pertussis* toxin IgG and *B. pertussis* FHA IgG) (Figure 2G–I and K–M). After adjustment for gestational age and sex in multivariable linear regression models, the associations between monocyte and MDSC levels and vaccine titers remained significant for *B. pertussis* toxin IgG and *B. pertussis* FHA IgG (adj. *p*-values 0.07, 0.008, and 0.004 for monocytes and *H. influenzae* type B IgG, *B. pertussis* toxin IgG, *B. pertussis* FHA IgG, and adj. *p*-values 0.19, 0.001, and 0.007 for MDSC and *H. influenzae* type B IgG, *B. pertussis* toxin IgG, *B. pertussis* FHA IgG. Preterm girls with early antibiotics had higher proportions of monocytes and MDSC than boys with early antibiotics (Figure 2J + N). An interaction analysis between sex and early antibiotic exposure showed a significant interaction for monocytes (*p* = 0.04), whereas no significant interaction was observed for MDSC (*p* = 0.6).

### The microbiome of antibiotic-exposed infants is dominated by *Klebsiella* and lacks *Bifidobacteria*

**Figure 3.**
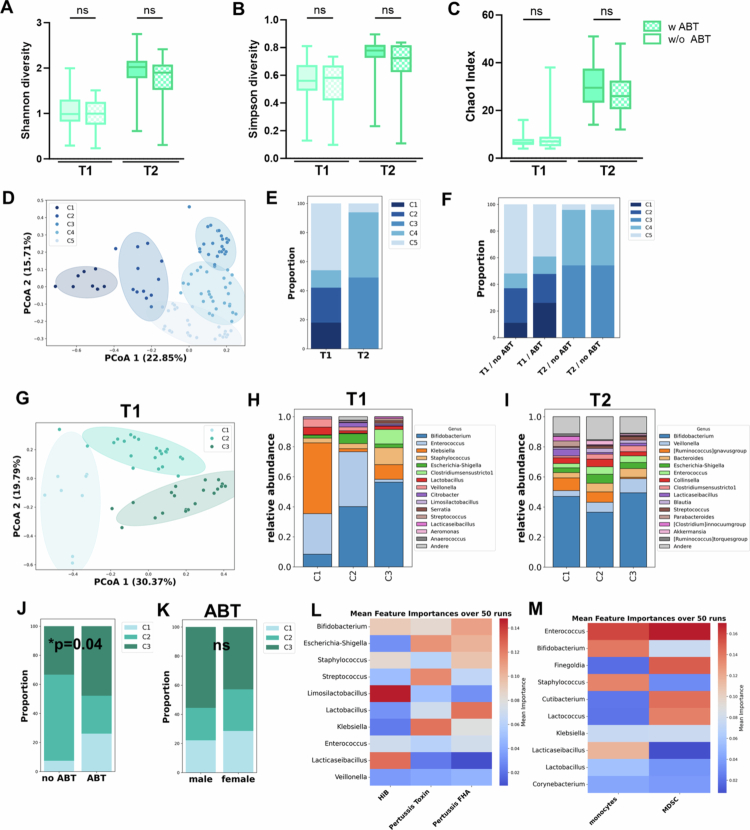
Microbiome composition in preterm infants without and with early antibiotic exposure. Stool samples were collected from preterm infants without and with antibiotic exposure in the first week of life at postnatal day 14 (T1, *n* = 50) and at a corrected age of four months (T2, *n* = 49) and 16S rRNA sequencing was performed. (A–C) Boxplots showing the *α*-diversity as calculated by the Shannon (A) and Simpson (B) index as well as the richness as estimated by the Chao1 index in preterm infants without (filled boxes, *n* = 27 at T1, *n* = 24 at T2) and with (checked boxes, *n* = 23 at T1, *n* = 25 at T2) early antibiotic exposure at both timepoints. ns not significant, Kruskal–Wallis test, and Dunn's multiple comparison test. (D–F) Principal coordinate analysis (PCoA) based on Bray–Curtis distance was plotted and a hierarchical clustering (*n* = 5 clusters) on all PCoA coordinates was performed. The cluster number was determined using a previously created dendrogram. (D) Principal coordinate analysis (PCoA) based on Bray–Curtis distance with hierarchical clustering (*n* = 5 clusters). (E + F) The stacked bar plots show the distribution of the five community types (C1–C5) identified in hierarchical clustering depending on the time point of sampling (E) and depending on whether infants were exposed to early antibiotics (F). (G–K) Principal coordinate analysis (PCoA) based on Bray–Curtis distance was plotted for the samples collected at T1 and a hierarchical clustering (*n* = 3 clusters) on all PCoA coordinates was performed. The cluster number was determined using a previously created dendrogram. (G) Principal coordinate analysis (PCoA) based on Bray–Curtis distance with hierarchical clustering (*n* = 3 clusters). (H + I) The stacked bar plots show the relative abundances of the 15 most common taxa in the three community types (C1–C3) identified in hierarchical clustering at T1 (H) and at T2 (I). (J) The stacked bar plot shows the distribution of the three community types (C1–C3) at T1 depending on whether infants were exposed to early antibiotics. (K) The stacked bar plot shows the distribution of the three community types at T1 in infants exposed to early antibiotics, depending on their sex. *p < 0.05, ns not significant, PERMANOVA. (L + M) Two random forest regressors with multi-output were trained to predict vaccine titers and innate immune cells (monocytes and MDSC) at a corrected age of 4 months and the mean feature importance was determined over 50 runs. The heatmaps shows the feature importance of the ten most predictive features for all three vaccine titers (L) and both innate immune cell types (M) divided according to their importance for each of the three titers/two immune cell types.

Regarding the microbiome, there were no differences in either Shannon or Simpson diversity or in the Chao 1 between infants with and without early antibiotic exposure in unadjusted analyses (Figure 3A–C) or after adjustment for known factors influencing the neonatal gut microbiome, including gestational age, sex, and delivery mode, neither at T1 nor at T2. When considered separately by sex, there was a tendency toward reduced diversity in antibiotic-exposed girls compared to unexposed girls at T1 (Supplementary figure 5A–C, interaction between antibiotic exposure and sex in adjusted regression models at T1: *p* = 0.09 for Shannon, *p* = 0.26 for Simpson, and *p* = 0.20 for Chao1, and at T2: *p* = 0.10 for Shannon, *p* = 0.08 for Simpson, and *p* = 0.21 for Chao1). Model-based adjusted effect plots are shown in Supplementary Figure 5D–I. To assess differences in microbial community composition between groups, beta diversity was calculated using the Bray–Curtis dissimilarity metric based on genus-level relative abundances and visualized by Principal Coordinates Analysis (PCoA). To identify subgroups based on microbiome profiles, hierarchical clustering was applied to all PCoA coordinates (Figure 3D). A comparison of the different community types at the two timepoints showed that community types 1, 2, and 5 (C1, C2, and C5) were mainly present at T1, while community types 3 and 4 (C3, C4) were predominantly found at T2 (Figure 3E). Neither at T1 nor at T2 were there any differences in the occurrence of the community types between infants with and without early antibiotic exposure (Figure 3F).

Clustering only on the samples taken at T1 resulted in three distinct community types (Figure 3G). Differential abundance testing using ANCOM-BC identified several genera that differed significantly between clusters, including *Veillonella, Bifidobacterium, Klebsiella, Clostridium sensu stricto, Escherichia–Shigella, Limosilactobacillus,* and *Enterococcus* (Supplementary Table 2). These genera were also among the most abundant taxa and are visualized in the stacked bar plots in Figure 3H. Community type 1, which was dominated by *Klebsiella* and had only a very low abundance of *Bifidobacteria* was observed more often in infants with antibiotic exposure. In contrast, community type 2, with high abundances of *Bifidobacteria* and *Enterococcus*, was more prevalent in infants without antibiotic exposure (Figure 3J). In the group of infants exposed to early antibiotics, there were no significant differences in the composition of community types between girls and boys (Figure 3K). Interestingly, at T2, the microbial compositions of the three clusters appeared more similar than at T1, indicating a convergence of the community profiles over time (Figure 3I). However, differential abundance analysis still identified taxa that differed significantly between clusters, including *Lactobacillus, Atopobium, Lacticaseibacillus, Akkermansia, Rombboutsia, Sellimonas, Lactobacillus*, and *Klebsiella* (Supplementary Table 3). To determine which taxa may be predictive of vaccine titers, a random forest regressor model with multi-output was trained and the mean feature importance was determined over 50 runs. As reported before, *Bifidobacteria* were among the most predictive genera for all vaccine titers.^21^ The top ten taxa predicting vaccine titers are shown in Figure 3L. Prediction of monocyte and MDSC levels identified the top ten taxa largely overlapping with those found for antibody titer prediction (Figure 3M). To assess the robustness of the identified microbial predictors, random forest models were additionally trained, including gestational age, sex, and delivery mode as clinical covariates. The taxa identified as most predictive for vaccine titers remained largely unchanged (Supplementary Figure 6A + B). Gestational age was also among the strongest predictors of vaccine titers, consistent with the known association between prematurity and reduced vaccine responses.

### Sex-dependent differences in plasma metabolites between preterm infants without and with early antibiotic exposure

**Figure 4.**
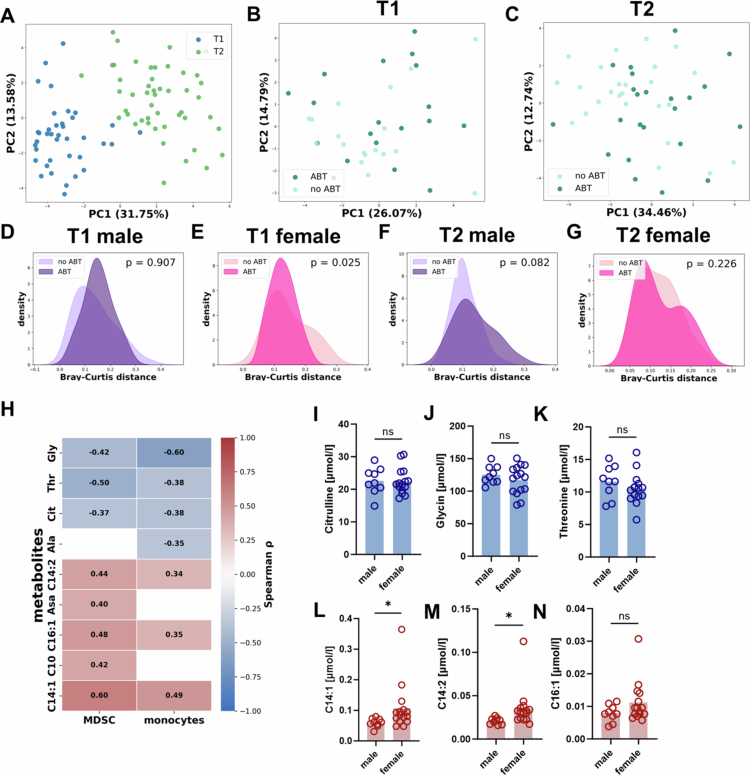
Plasma metabolites in preterm infants without and with early antibiotic exposure. EDTA plasma samples were collected from preterm infants without and with antibiotic exposure in the first week of life at postnatal day 14 (T1, *n* = 38) and at a corrected age of four months (T2, *n* = 49) and metabolite concentrations were determined by MS/MS. Principal component analysis (PCA) was plotted two-dimensional based on the first two principal components for all samples (A) or for samples collected at T1 (B) or T2 (C) separately. The blue dots symbolize samples taken on T1, the green dots symbolize samples taken on T2. Light turquoise dots symbolize samples from preterm infants without early antibiotic exposure, dark turquoise dots symbolize samples from preterm infants with early antibiotic exposure. (D–G) Bray-Curtis distances were determined and plotted pairwise in histograms for male (violet histograms, D + F) and female (pink histograms, E + G) preterm infants without and with antibiotic exposure at the two sampling timepoints. A PERMANOVA was performed for each comparison pair. (H) Correlation analyses between metabolite concentrations and the innate immune cells monocytes and MDSC were done using Spearman's rank correlation. Multiple testing was performed using the Benjamini–Hochberg procedure. The heatmap shows all significant correlations between plasma metabolites and MDSC (left column) and monocytes (right column). Positive correlations are colored in red, negative correlations are colored in blue. (I–N) The scattered bar charts show metabolite levels of metabolites that were shown to correlate with the levels of monocytes and MDSC in the plasma of infants with early antibiotic exposure stratified by sex. The blue diagrams (I–K) show metabolites with negative correlation with monocytes and MDSC, the red diagrams (L–N) show metabolites with positive correlation with monocytes and MDSC. * *p* < 0.05, ns not significant, unpaired *t*-test or Mann–Whitney test.

Lastly, we explored levels of certain metabolites (19 amino acids, 21 acylcarnitines, and succinate) in the plasma of study participants. The PCA of the metabolite compositions of all measured plasma samples showed a clear separation of the samples taken at T1 and at T2 (*p* = 0.001 in PERMANOVA, Figure 4A). Neither at T1 (*p* = 0.24 in PERMANOVA) nor at T2 (*p* = 0.09 in PERMANOVA) was there a clear separation in the PCA depending on early antibiotic exposure (Figure 4B + C). No differences were found in Bray-Curtis distances between antibiotic-exposed and non-exposed preterm boys at T1 (*p* = 0.88 in PERMANOVA, Figure 4D) and between antibiotic-exposed and non-exposed preterm boys (*p* = 0.09 in PERMANOVA, Figure 4F) and girls (*p* = 0.23 in PERMANOVA, Figure 4G) at T2. However, at T1, the metabolite profiles of preterm girls with early antibiotic exposure differed significantly from those of preterm girls without antibiotic exposure (Figure 4E, *p* = 0.03 in PERMANOVA).

In Spearman's rank correlation, a positive correlation with both monocytes and MDSC was found for the acylcarnitines C14:1 (tetradecenoylcarnitin), C14:2 (tetradecadienoylcarnitin), and C16:1 (palmitoleylcarnitin), and a negative correlation with both immune cell types was found for the amino acids glycin, threonine, and citrulline (Figure 4H). A comparison of the levels of these metabolite concentrations in the plasma of male and female preterm infants exposed to early antibiotics showed that female infants had elevated levels of C14:1 and C14:2 compared to male infants (Figure 4L + M), while the other metabolites did not differ significantly between sexes (Figure 4I–K + N).

## Discussion

The main aim of this study was to investigate the effect of early antibiotic exposure on the antibody response to vaccination at a corrected age of four months in preterm infants at a corrected age of four months. Consistent with previous studies in mice and term-born infants,^20^ ^,^ ^21^ we found decreased vaccine titers against *pertussis* and *H. influenzae type B* also in preterm infants exposed to antibiotics during their first week of life compared to non-exposed infants. In addition to absolutely reduced vaccine titers, Ryan et al. reported that infants with early antibiotic exposure were more likely to have vaccine titers below the protective threshold at seven months of age.^21^ The seroprotective thresholds for pertussis FHA, pertussis toxin, and HiB PRP were given in the study as 5000 mIU/ml, 5000 mIU/ml, and 150 ng/ml, respectively. On average, the vaccine titers against HiB in our study infants investigated at four months' corrected age were five to ten times higher than these thresholds. Several previous studies have also reported highly variable antibody titers across different cohorts.^42-44^ Reasons for these differences in HiB titer levels remain unclear. A plausible explanation is that antibody titers in our cohort were quantified at an earlier post-vaccination time point than in the study by Ryan et al. (age of four vs. seven months). Overall, only a small number of infants in our cohort had antibody titers below the respective protective thresholds, suggesting limited clinical relevance of our findings. Nevertheless, they suggest that early antibiotic exposure may influence immune development. Larger studies with longer follow-up are needed to better assess the true clinical impact.

Exploratory sex-stratified analyses suggested a stronger reduction in vaccine titers in girls. Interestingly, various studies suggest that adult women develop a stronger immune response to vaccinations.^40^ A similar trend was observed in newborns with a higher response to Diphteria-toxoid, Meningococcal and Pneumococcal vaccines in girls.^45^ Furthermore, it has been known for a long time that male preterm infants have a higher risk of adverse outcomes than girls.^46^ ^,^ ^47^ These differences are attributed to sex-dependent differences in immune response, neural and neurovascular development, lung development, and surfactant production.^46^ ^,^ ^48-50^ In our control group, we saw no significant differences in the level of vaccine titers measured between male and female preterm infants. The duration of antibiotic exposure also did not differ between boys and girls (not shown). The greater decrease in vaccine titers after antibiotic exposure, therefore, indicates a higher susceptibility of female infants to early antibiotic therapy. Women have been shown to develop adverse drug reactions more frequently than men, and it has been shown that various medications lead to higher blood levels and prolonged elimination times in women.^51^ Thus, it could be speculated that antibiotics have prolonged and thus stronger effects in preterm girls than boys. However, given the exploratory nature of the sex-stratified analyses and the limited sample size, these findings should be interpreted with caution.

The modulation of immune development by external influences during the perinatal period plays an important role in the development of later immune-associated diseases. Compared to term newborns, preterm infants exhibit differences in immune cell composition, function, and development.^52^ While we observed hardly any effect of early antibiotic exposure on immune cell composition in the whole cohort, we found a negative correlation between the innate immune cell types monocytes and MDSC and all three vaccine titers. We also noted elevated levels of both monocytes and MDSC in antibiotic-treated preterm girls compared to antibiotic-treated preterm boys at a corrected age of four months. In an animal model it was shown that preventing monocyte recruitment to the lymph nodes after vaccination significantly improved the cellular and humoral immune response to vaccination^53^ and in HIV patients, high levels of classical/inflammatory monocytes were associated with a poorer immune response to influenza vaccination and suppressed antigen-specific CD4 T-cell proliferation *in vitro.* ^54^ Unfortunately, we did not differentiate between classical and non-classical monocytes in our study, but in general, newborns have increased proportions of classical monocytes,^55^ which could explain the negative correlation with vaccine titers that we observed. MDSC are suppressive acting innate immune cells that are present in significantly higher numbers in newborns than in adults and control inflammation in the perinatal period.^56-60^ Furthermore, it has been shown that they contribute to a poorer response to vaccinations,^61^ ^,^ ^62^ which is consistent with our observation that they are negatively correlated with antibody titers in preterm infants. Some studies have described sex-specific differences in the inflammatory activation and an accumulation of monocytes and MDSC.^63^ ^,^ ^64^ However, to our knowledge, it has not yet been investigated how antibiotics may influence these sex-specific differences. Together, the data presented here raise the hypothesis that a persistence of suppressively acting innate immune cells, which may be triggered by early antibiotic exposure, especially in preterm girls, contributes to an inhibition of the vaccine response. Further studies are needed to investigate this hypothesis and the underlying mechanisms in more detail.

Our findings regarding the influence of early antibiotic exposure on the microbiome of preterm infants are somewhat surprising. In contrast to other studies that showed profound effects of antibiotic therapy on the microbiome of newborns and preterm infants,^21^ ^,^ ^65^ ^,^ ^66^ we found no differences in alpha diversity, neither at T1 nor at T2, between antibiotic-exposed and non-exposed preterm infants. Only at postnatal day 14, samples from antibiotic-exposed infants differed in their community types from that of non-exposed infants with a higher proportion of infants belonging to a community type dominated by *Klebsiella* and having a very low abundance of *Bifidobacteria*. By the corrected age of four months, these differences had largely disappeared. Here, it has to be noted that all preterm infants in our cohort received probiotics (Infloran®, consisting of *B. bifidum* and *L. acidophilus*) until a postmenstrual age of 32 weeks. Similar to our findings, a recently published study found no differences in microbial diversity between probiotic-treated (same probiotic as in our cohort) preterm infants with and without antibiotic exposure, and a clear predominance of *Bifidobacteria* in the microbiome of probiotic-treated infants.^67^ The cited work additionally found that probiotic supplementation significantly reduced antibiotic resistance gene (ARG) prevalence and multi-drug resistant (MDR) pathogen load,^67^ suggesting that probiotics may partially mitigate the effects of antibiotics on the preterm microbiome and could therefore contribute to the observed findings.

In their aforementioned study, Ryan et al. showed that reduced vaccine titers in term newborns exposed to early antibiotics correlated with a reduced abundance of different *Bifidobacteria* strains and that administration of *Bifidobacteria* to germ-free mice significantly improved their immune response to vaccination.^21^ This is consistent with our results from the random forest regressor models, which show that *Bifidobacteria* are among the most important predictors of both vaccine titers and the levels of monocytes and MDSCs. As all infants in our cohort received *Bifidobacteria* as a probiotic, this may also help explain why overall antibody titers were more frequently within the protective range compared with the study by Ryan et al. However, despite universal probiotic supplementation in our cohort, differences in vaccine responses associated with early antibiotic exposure were still observed, suggesting that the probiotics used in our cohort may not have been sufficient to completely overcome early-life perturbations. Future studies are needed to determine whether more targeted microbiome interventions could further enhance vaccine responses in this vulnerable population.

It has been widely reported that metabolism has a decisive influence on immune responses (so-called immunometabolism)^68^ and, in particular, vaccine responses.^69^ For example, it was shown that microbiome disruption by antibiotics in newborns leads to altered tryptophan metabolism, which in turn influenced immune development.^14^ ^,^ ^70^ Thus, we explored specific plasma metabolites in preterm infants with and without early antibiotic exposure at time points T1 and T2. We found no differences in the metabolite profile between antibiotic-exposed and non-exposed infants within the limits of our approach, assessing only selected metabolites.

When male and female preterm infants were analyzed separately, exploratory analyses suggested that preterm girls showed significant changes in the metabolite profile at T1 if they had received early antibiotics, whereas this was not seen in preterm boys. The mechanism underlying the sex-specific differences in the metabolite profile after early antibiotic exposure remains unclear. It can only be speculated that hormonal differences and genetic differences in metabolism probably play a role here.

Exploratory correlation analyses revealed associations between the acylcarnitines C14:1, C14:2, and C16:1, as well as the amino acids glycin, threonine and citrulline with MDSC and monocytes. Of these, only C14:1 and C14:2 were elevated, albeit to a very low level overall, in antibiotic-exposed preterm girls compared to boys and could therefore represent a link between metabolite profile and elevated monocyte/MDSC levels as well as reduced vaccine titers in preterm girls after early antibiotic exposure. However, these findings should be interpreted cautiously, given the exploratory nature of our analyses. To our knowledge, nothing is known about the immunological effects of these two fatty acids. For other monounsaturated and polyunsaturated fatty acids it has been shown *in vitro* that they suppress the production of pro-inflammatory cytokines in monocytes^71^ and that they induce a immunosuppressive phenotype in tumor associated macrophages (TAMs).^72^ Similarly, monounsaturated and polyunsaturated fatty acids, but not saturated fatty acids, appear to induce a regulatory, MDSC-like phenotype in bone marrow cells.^73^ It remains unclear whether the elevated levels of C14:1 and C14:2 in preterm girls exposed to antibiotics contribute to the induction of a more anti-inflammatory phenotype in innate immune cells, which may then lead to suppression of the immune response to vaccination. Further *in vitro* and *in vivo* studies are needed to investigate the link between antibiotics, metabolome, and immune response in preterm infants in more detail.

A major strength of this study is its prospective design, enabling targeted data collection and excluding the possibility of recall bias. Furthermore, to our knowledge, this is the first study investigating the impact of early antibiotic exposure on vaccine responses in a preterm cohort. Another strength lies in the comprehensive approach, as we assessed multiple outcome domains—including vaccine responses, immune cell profiles, the gut microbiome, and plasma metabolites—and integrated these data to provide a more holistic view of the interactions between antibiotics and the host. The main limitation of our study is that not all samples could be collected from every participant, resulting in an incomplete data set. The low sample volume we could obtain from these infants constrained the number of vaccine titers that could be analyzed, and immune cell characterization was limited to phenotypic profiling without functional assays. Potential confounding factors such as later antibiotic exposures and mode of delivery were not explored in depth. With regard to analysis techniques, another limitation is that microbiome analyses were restricted to 16S rRNA sequencing rather than shotgun metagenomics, and metabolomic profiling was limited to selected plasma metabolites without the inclusion of fecal metabolites. Furthermore, information on maternal vaccination status and information on postnatal antibiotic therapy to the mother or the infant after discharge was not collected and could thus not be considered in our analysis. Finally, the relatively small number of patients included in this study represents an important limitation, particularly for the machine learning analyses. However, to the best of our knowledge, this dataset represents the largest cohort reported so far investigating associations between gut microbiome composition, early antibiotic exposure, and vaccine responses in preterm infants. As the data were obtained from routine clinical care in a highly vulnerable patient population, the availability of samples was inherently limited. Importantly, the primary aim of the Random Forest models was not to develop a highly predictive model but to identify microbial taxa potentially associated with vaccine responses in an exploratory manner. To reduce the risk of overfitting, we applied several strategies suitable for small datasets, including repeated model training using 50 different random data splits, constrained hyperparameter tuning, and the use of Random Forest models, which employ bootstrap aggregation to increase model stability. Nevertheless, the findings should be interpreted cautiously and require confirmation in larger independent cohorts.

In summary, the present study demonstrates that, consistent with findings from cohorts of term newborns^21^ and animal models,^20^ early antibiotic exposure also affects vaccine responses in preterm infants. Extending the existing literature, we show for the first time that the impact of antibiotics on vaccine responses may differ between sexes. A potential underlying mechanism could involve antibiotic-induced alterations in the microbiome and metabolome of preterm infants, leading to the persistence of innate immune cells with suppressive properties that subsequently impair adaptive immune responses to vaccination. As this study establishes associations rather than causality, further investigations are needed to clarify the mechanistic links between early antibiotic exposure and immune response to vaccination.

## Supplementary Material

Supplementary Figure4_ABX and vaccination_revision_final.tif Supplementary Figure4_ABX and vaccination_revision_final.tif

Supplementary Figure7_ABX and vaccination_revision_final.tif Supplementary Figure7_ABX and vaccination_revision_final.tif

Supplementary Figure3b_ABX and vaccination_revision_final.tif Supplementary Figure3b_ABX and vaccination_revision_final.tif

Supplementary Figure1_ABX and vaccination_revision_final.tif Supplementary Figure1_ABX and vaccination_revision_final.tif

Supplementary Figure6b_ABX and vaccination_revision_final.tif Supplementary Figure6b_ABX and vaccination_revision_final.tif

Supplementary Figure3a_ABX and vaccination_revision_final.tif Supplementary Figure3a_ABX and vaccination_revision_final.tif

Supplementary Figure5a_ABX and vaccination_revision_final.tif Supplementary Figure5a_ABX and vaccination_revision_final.tif

Supplementary Figure5b_ABX and vaccination_revision_final.tif Supplementary Figure5b_ABX and vaccination_revision_final.tif

manuscript early antibiotics and vaccination_Supp tables_final.docx manuscript early antibiotics and vaccination_Supp tables_final.docx

Supplementary Figure2_ABX and vaccination_revision_final.tif Supplementary Figure2_ABX and vaccination_revision_final.tif

Supplementary Figure6a_ABX and vaccination_revision_final.tif Supplementary Figure6a_ABX and vaccination_revision_final.tif

## Data Availability

Sequencing data and metadata for all samples used in this study have been deposited in NCBI Sequence Read Archive (SRA) under accession number PRJNA1400095. The underlying code for this study is not publicly available for proprietary reasons.

## Abbreviations

ABT: antibiotic therapy
EDTA: ethylenediaminetetraacetic acid
ESI: electrospray ionization
FIA: flow injection analysis
FCS: fetal calf serum
FHA: filamentous hemagglutinin
HiB: *Haemophilus influenzae* B
Ig: immunoglobulin
MDSC: myeloid derived suppressor cells
MNC: mononuclear cells
MS/MS: tandem mass spectrometry
PBMC: peripheral blood mononuclear cells
Th: T helper
Treg: regulatory T cells
VLBW: very low birth weight
