# Supplementary figures and images for: Early antibiotic exposure and vaccine immune responses in preterm infants: potential sex-specific differences
Source: Gut Microbes. 2026 Jun 27;18(1):2694122. doi: 10.1080/19490976.2026.2694122 (PMC13313264; doi:10.1080/19490976.2026.2694122)

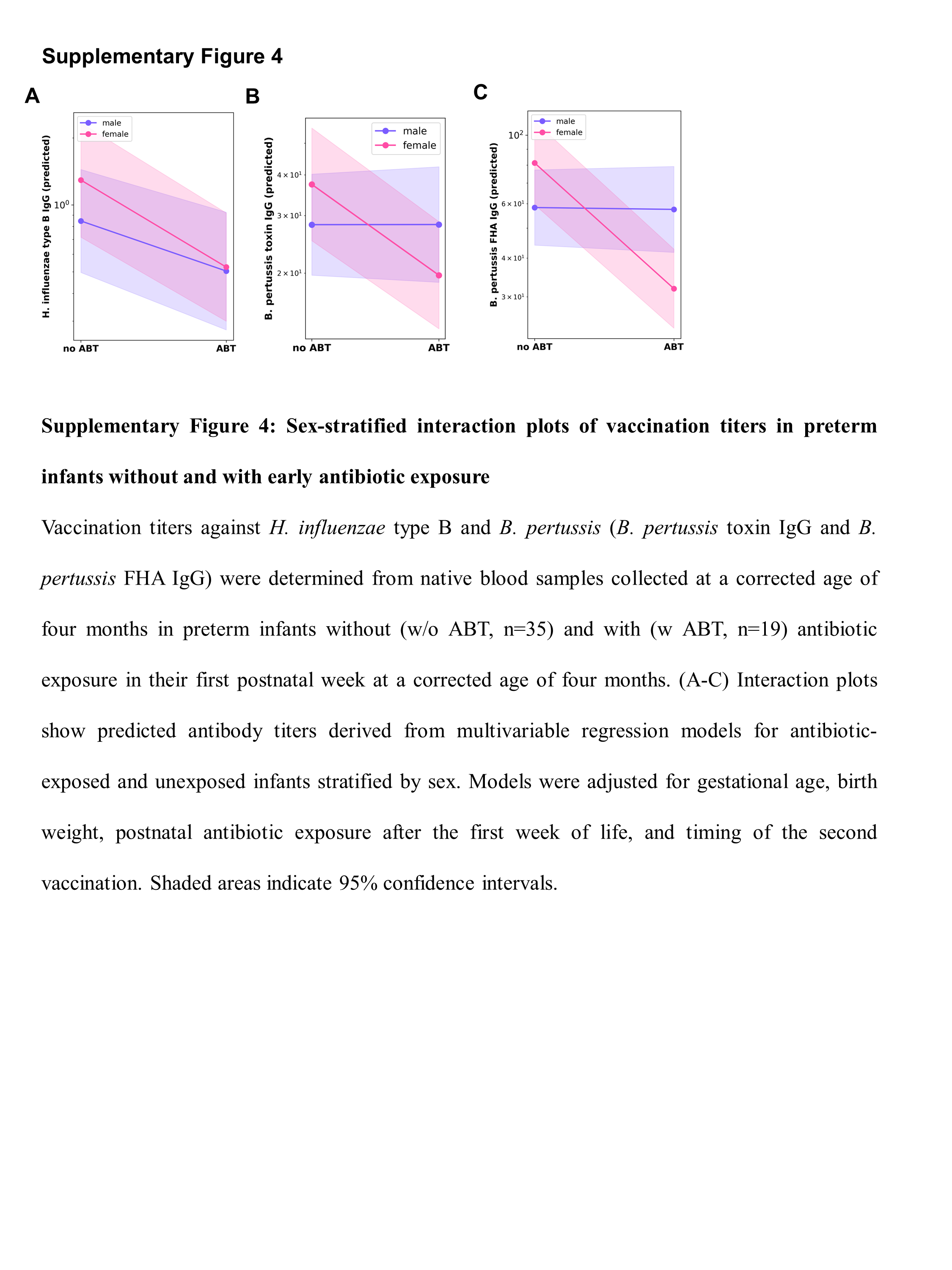

Supplement: Supplementary Figure4_ABX and vaccination_revision_final.tif [file KGMI_A_2694122_SM8239.tif]

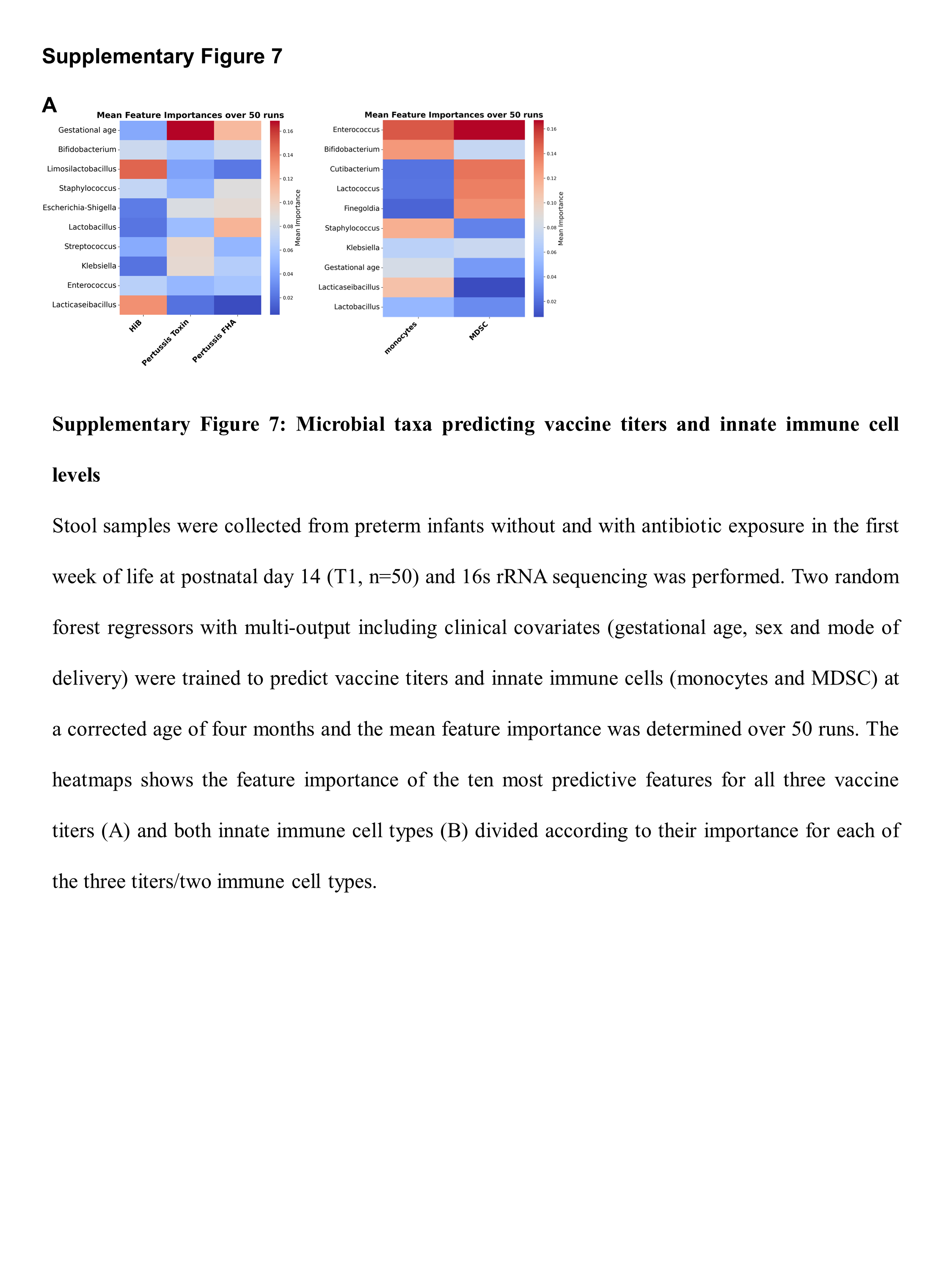

Supplement: Supplementary Figure7_ABX and vaccination_revision_final.tif [file KGMI_A_2694122_SM8229.tif]

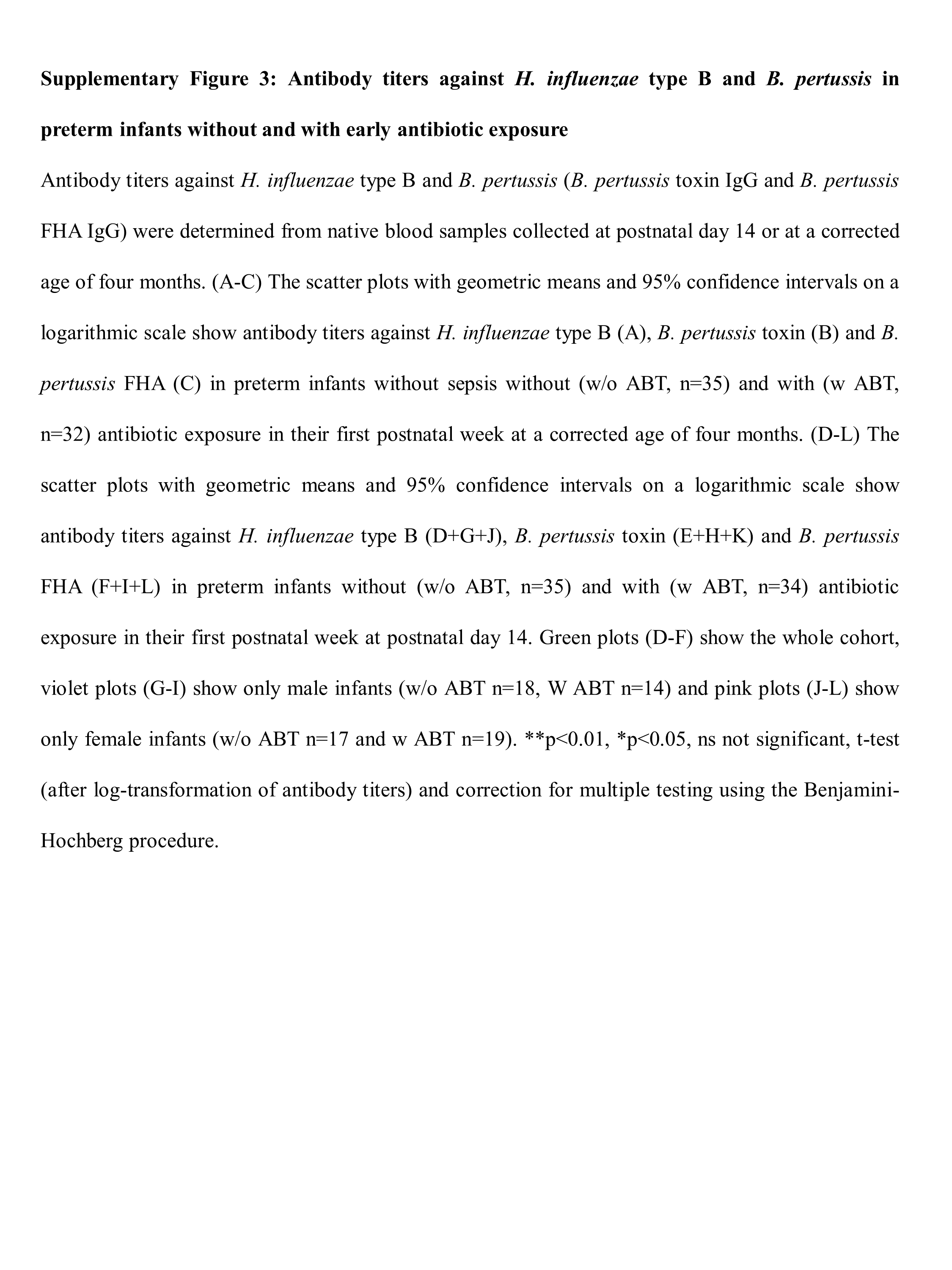

Supplement: Supplementary Figure3b_ABX and vaccination_revision_final.tif [file KGMI_A_2694122_SM8230.tif]

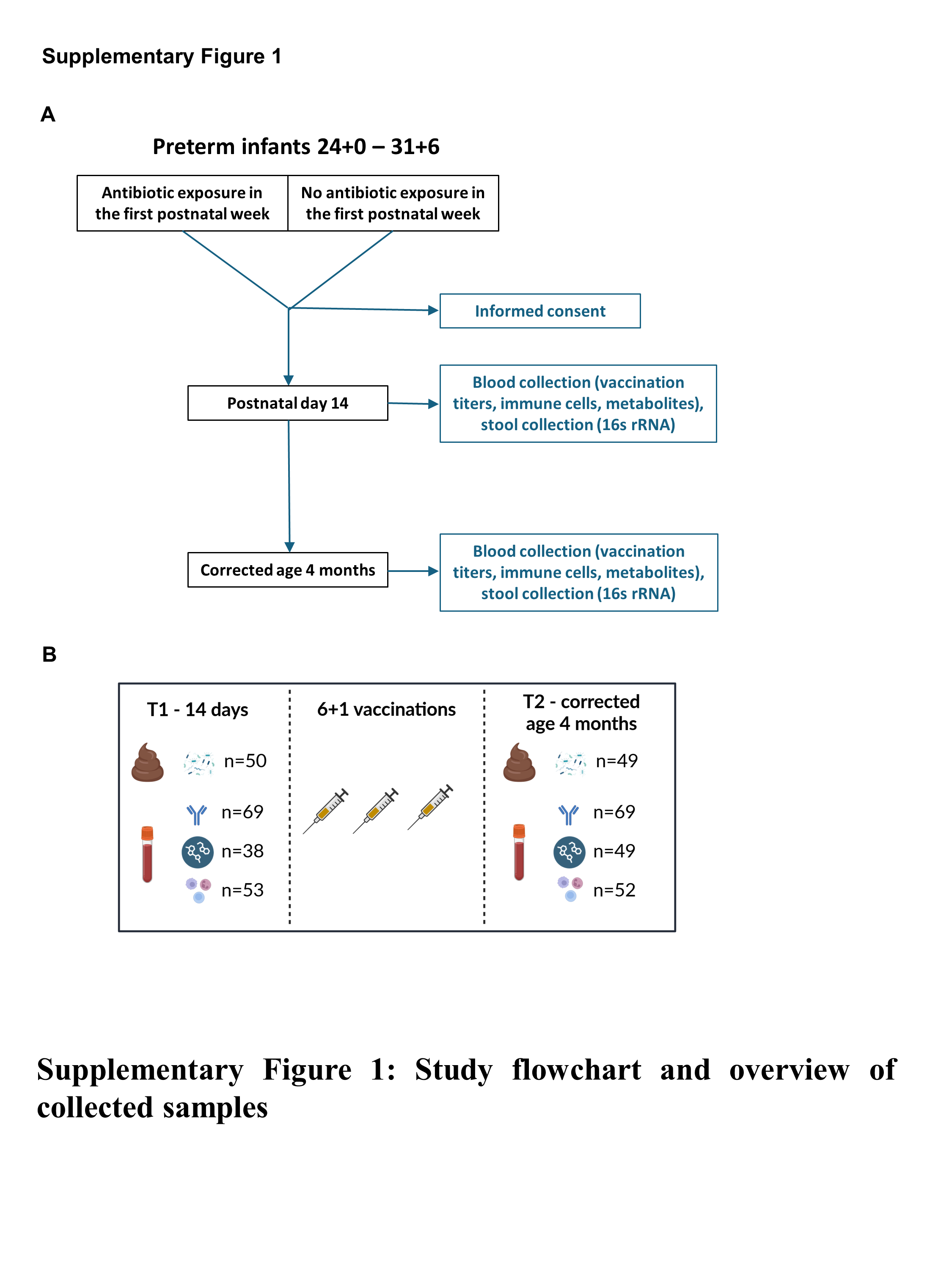

Supplement: Supplementary Figure1_ABX and vaccination_revision_final.tif [file KGMI_A_2694122_SM8231.tif]

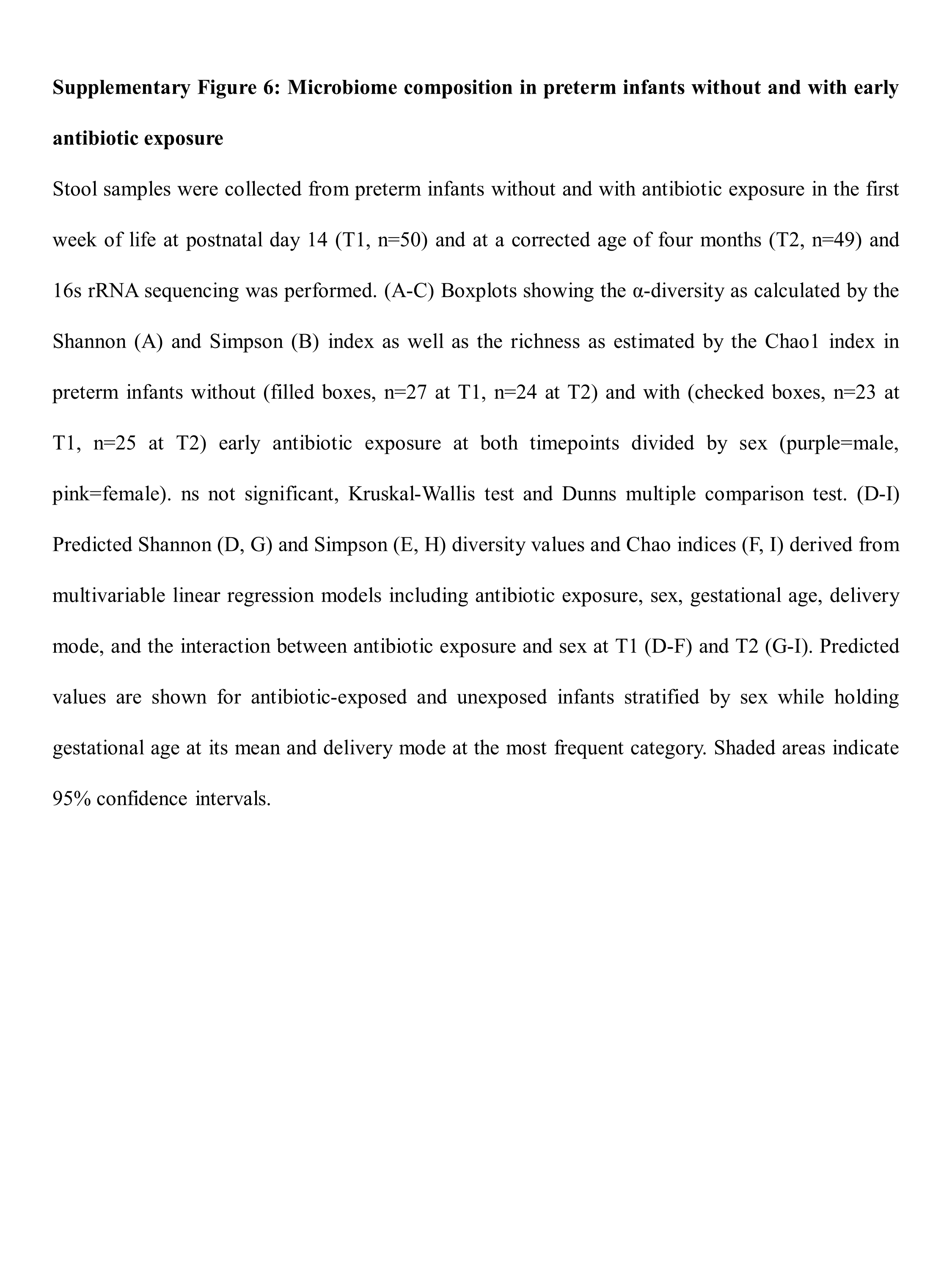

Supplement: Supplementary Figure6b_ABX and vaccination_revision_final.tif [file KGMI_A_2694122_SM8232.tif]

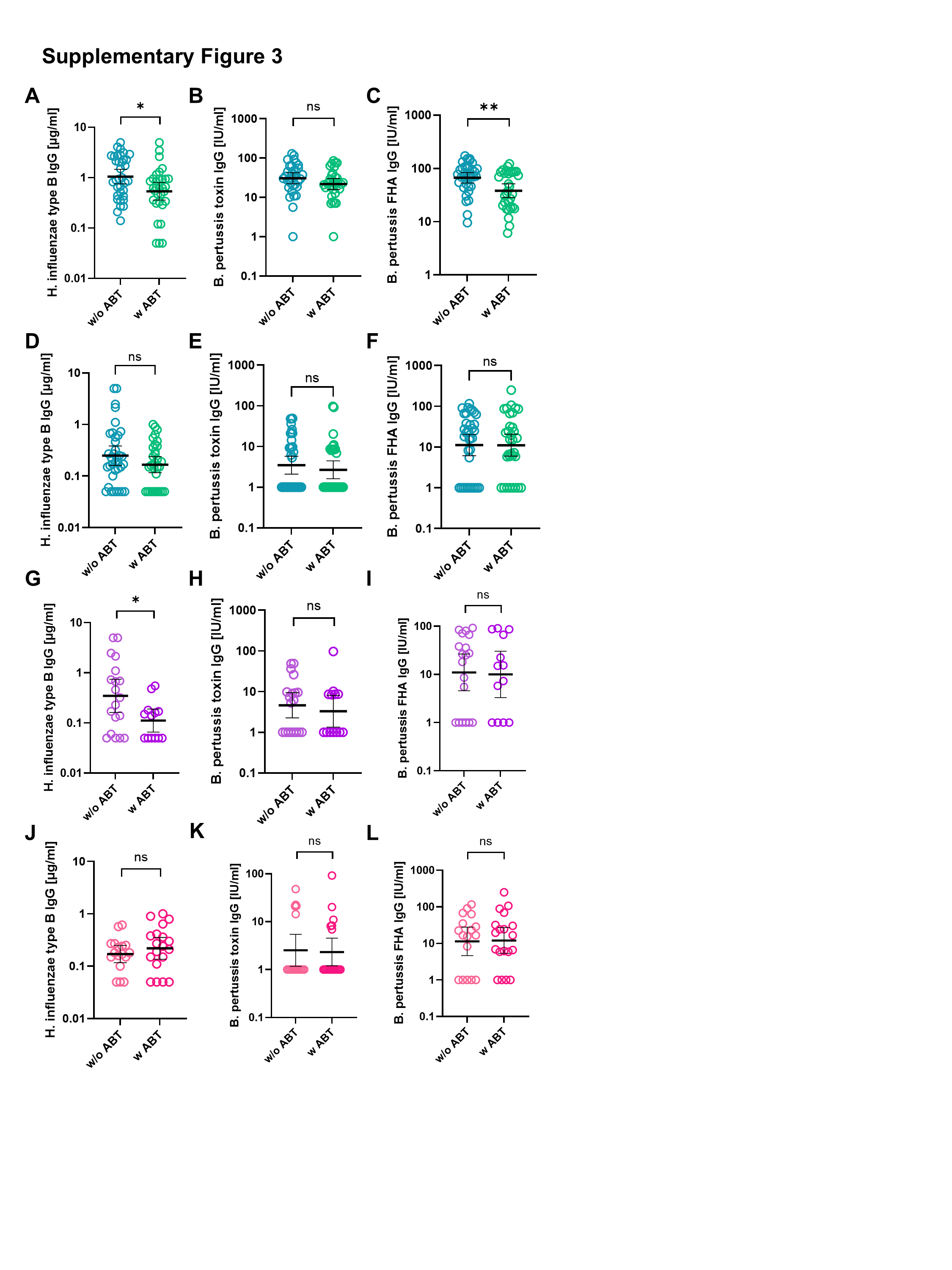

Supplement: Supplementary Figure3a_ABX and vaccination_revision_final.tif [file KGMI_A_2694122_SM8233.tif]

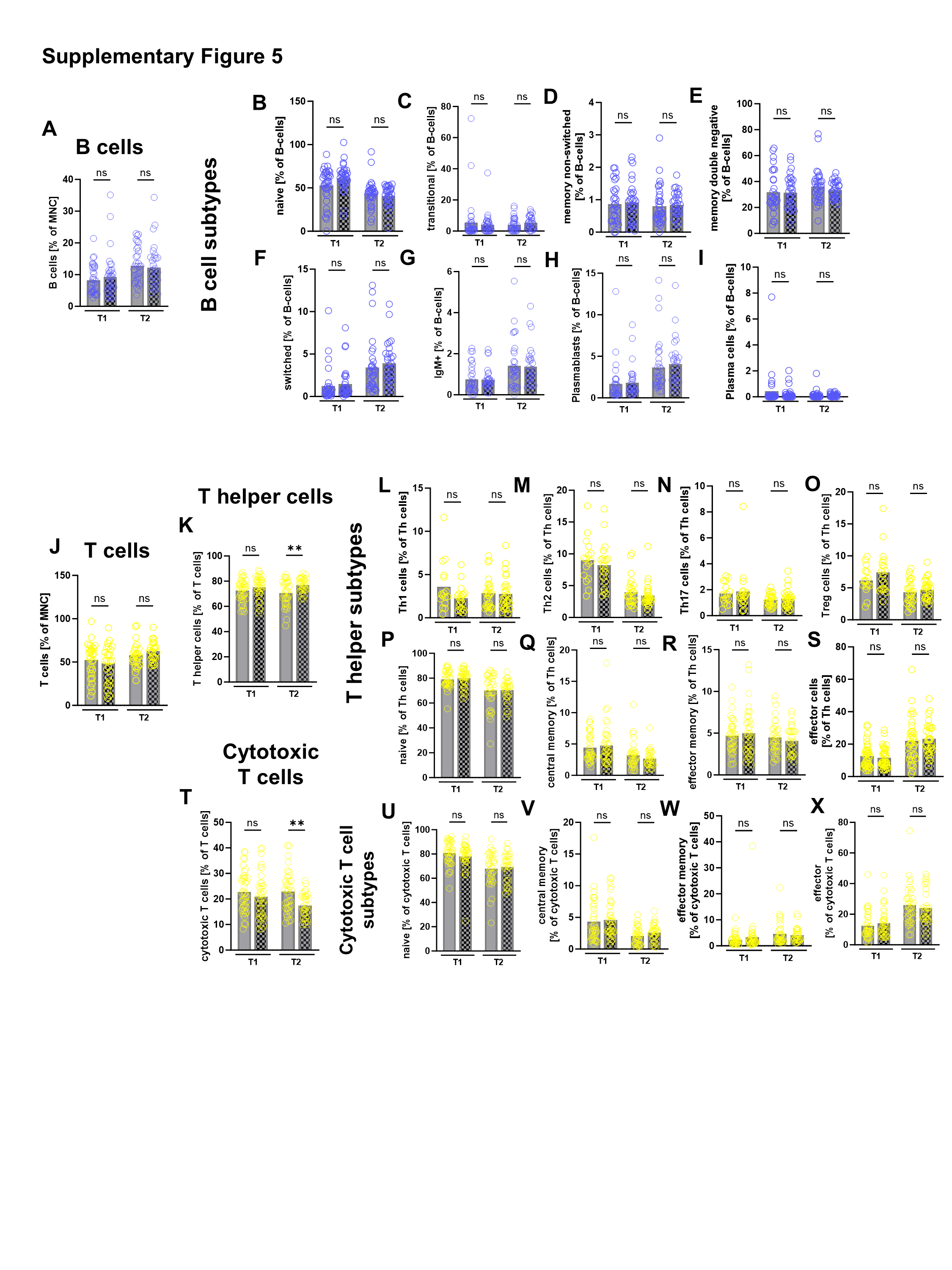

Supplement: Supplementary Figure5a_ABX and vaccination_revision_final.tif [file KGMI_A_2694122_SM8234.tif]

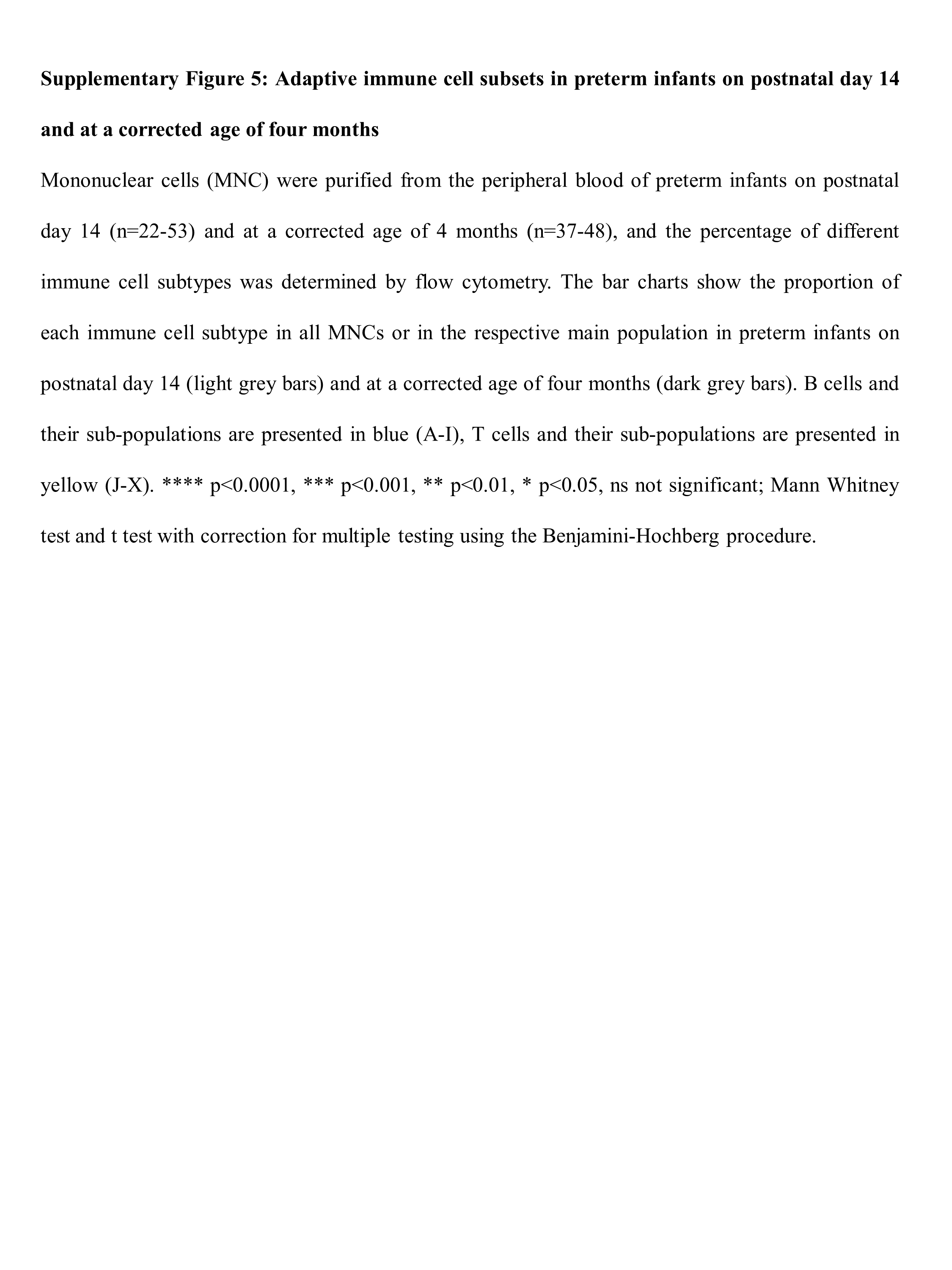

Supplement: Supplementary Figure5b_ABX and vaccination_revision_final.tif [file KGMI_A_2694122_SM8235.tif]

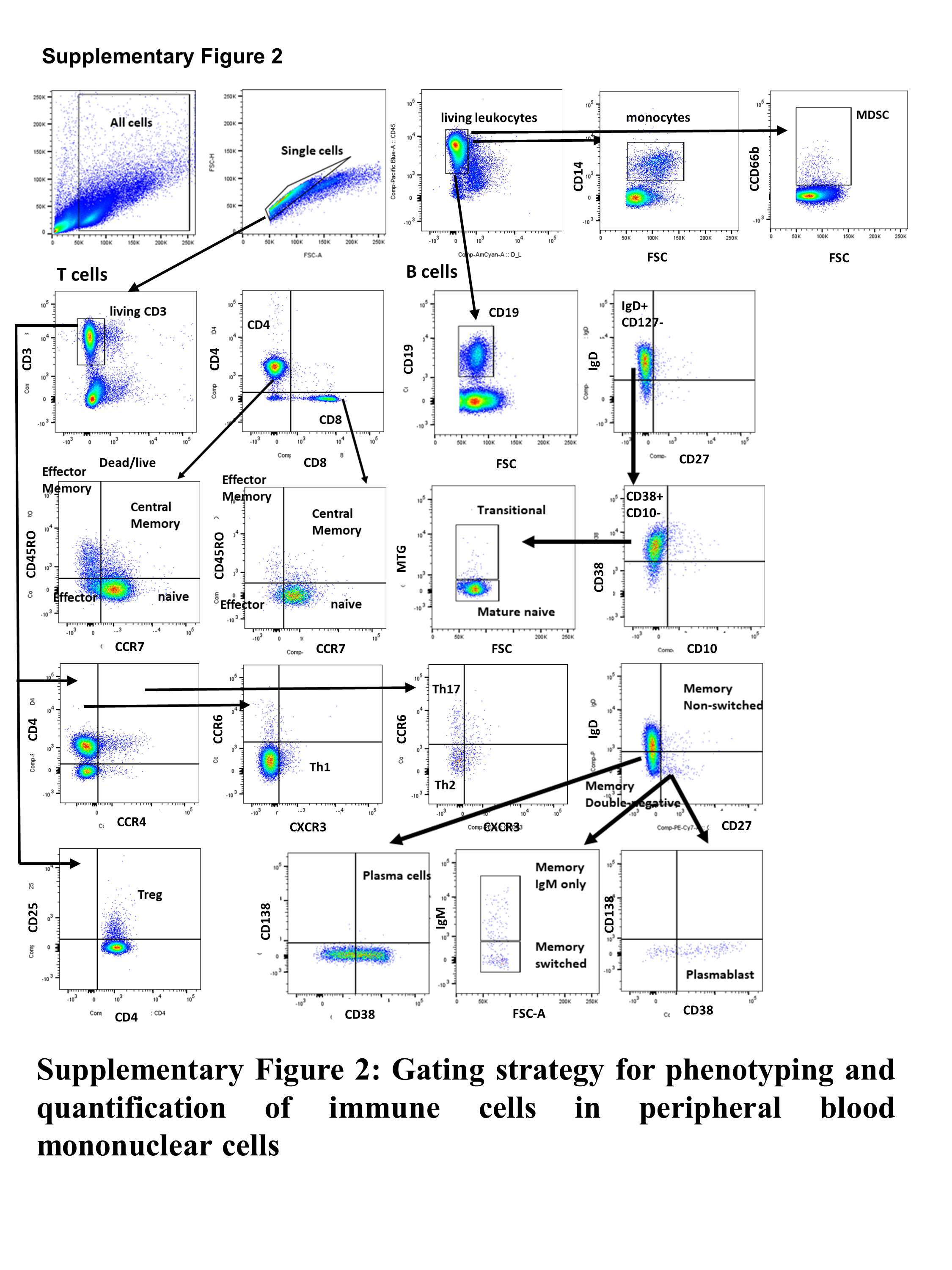

Supplement: Supplementary Figure2_ABX and vaccination_revision_final.tif [file KGMI_A_2694122_SM8237.tif]

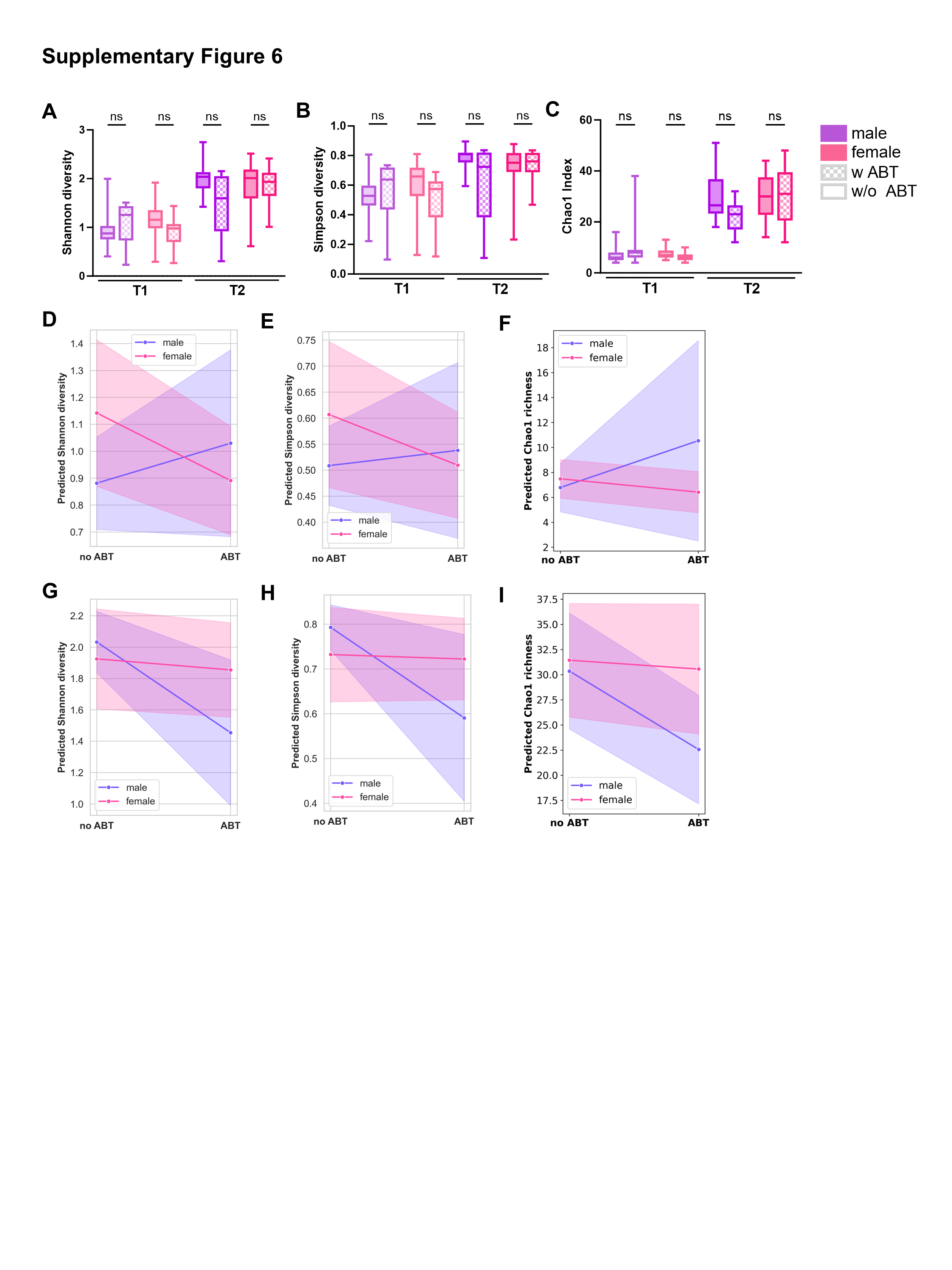

Supplement: Supplementary Figure6a_ABX and vaccination_revision_final.tif [file KGMI_A_2694122_SM8238.tif]
