# Supplementary material for: Early antibiotic exposure and vaccine immune responses in preterm infants: potential sex-specific differences
Source: Gut Microbes. 2026 Jun 27;18(1):2694122. doi: 10.1080/19490976.2026.2694122 (PMC13313264; doi:10.1080/19490976.2026.2694122)
Supplement: manuscript early antibiotics and vaccination_Supp tables_final.docx [file KGMI_A_2694122_SM8236.docx]

**Supplementary table 1: Significant changes in B and T cell subpopulations over time**

| Cell type | β: T2 vs. T1 (95% CI) | Adj. p-value |
| --- | --- | --- |
| T-cells | 14.5 (5.1-23.8) | <0.01 |
| Th2 cells (% of T helper cells) | -4.2 (-6.3- -2.0) | <.0.01 |
| T_reg_ (% of T helper cells) | -2.4 (-4.1- -0.6) | 0.02 |
| Central Memory CD4^+^ T cells | -1.9 (-3.3- -0.5) | 0.02 |
| Naive CD4^+^ T cells | -9.9 (-15.5- -4.4) | <0.01 |
| Effector CD4^+^ T cells | 11.2 (5.4-16.9) | <0.01 |
| Central Memory CD8^+^ T cells | -1.9 (-3.4- -0.5) | 0.02 |
| Effector CD8^+^ T cells | 8.4 (2.4-14.4) | 0.02 |
| Naive B cells | -20.8 (-30.0- -11.7) | <0.01 |
| Switched B cells | 2.7 (1.4-4.0) | <0.01 |
| Plasmablasts | 2.5 (1.1-3.9) | <0.01 |

**Supplementary table 2: Differentially abundant genera between microbiome community types at T1**

| Genus | Comparison | logFC | Adj. p-value |
| --- | --- | --- | --- |
| *Veillonella* | C3 vs. C1 | -5.24 | **<0.001** |
| *Veillonella* | C2 vs. C1 | -4.99 | **<0.001** |
| *Bifidobacterium* | C3 vs. C1 | 3.47 | **<0.01** |
| *Klebsiella* | C2 vs. C1 | -2.68 | **<0.01** |
| *Clostridium sensu stricto* | C3 vs. C1 | 4.21 | **0.02** |
| *Escherichia-Shigella* | C2 vs. C1 | -2.32 | **0.04** |
| *Bifidobacterium* | C2 vs. C1 | 2.20 | **0.04** |
| *Limosilactobacillus* | C2 vs. C1 | -2.26 | **0.04** |
| *Enterococcus* | C3 vs. C1 | -2.13 | **0.05** |

**Supplementary table 3: Differentially abundant genera between microbiome community types at T2**

| Genus | Comparison | logFC | Adj. p-value |
| --- | --- | --- | --- |
| *Lactobacillus* | C3 vs. C1 | 4.45 | **0.02** |
| *Atopobium* | C2 vs. C1 | -4.15 | **0.02** |
| *Lacticaseibacillus* | C2 vs. C1 | -2.78 | **0.02** |
| *Akkermansia* | C2 vs. C1 | 2.75 | **0.02** |
| *Romboutsia* | C2 vs. C1 | -2.66 | **0.02** |
| *Sellimonas* | C2 vs. C1 | 2.66 | **0.02** |
| *Lactobacillus* | C2 vs. C1 | 2.83 | **0.03** |
| *Klebsiella* | C2 vs. C1 | 1.88 | **0.03** |
